# Simultaneous measurement of water transport across the blood–brain and blood–CSF barrier in the human brain with arterial spin labeling MRI

**DOI:** 10.1177/0271678X261429042

**Published:** 2026-03-17

**Authors:** Léonie Petitclerc, Helena Durrant, Lydiane Hirschler, Lena Václavů, Matthias J.P. van Osch

**Affiliations:** 1 C.J. Gorter MRI Center, Department of Radiology, Leiden University Medical Center, Leiden, Netherlands; 2 Leiden Institute for Brain and Cognition (LIBC), Leiden, Netherlands

**Keywords:** Arterial spin labeling, blood–brain barrier, blood–CSF barrier, brain clearance, glymphatics, MRI, water exchange

## Abstract

Previously, water exchange across the blood–brain barrier (BBB) and blood–cerebrospinal barrier (BCSFB) was assessed by multi-delay, multi-echotime (TE) arterial spin labeling, albeit in separate acquisitions with different settings. In this study, we present a protocol for simultaneous measurement of BBB and BCSFB water exchange using a multi-TE 3D-GRASE acquisition combined with T_2_-preparation. We evaluate several modeling approaches, comparing two- and three-compartment models to estimate water exchange rates from blood to gray matter (*K_bl_*_ → GM_), and to CSF (*K_bl_*_ → CSF_). ASL signal was consistent with expectations: at early time points, signal decayed rapidly across TEs in blood and GM, whereas at later time points decay was slower, consistent with label accumulation in CSF. All models yielded CBF and ATT values consistent with literature. *K_bl_*_ → CSF_ was highest in the choroid plexus (≈1.75 × 10^−2^ s^–1^), with lower values in GM and subarachnoid space (≈1.4 × 10^−2^ s^–1^), and white matter (≈1.2 × 10^−2^ s^–1^) ROIs. In contrast, *K_bl_*_ → GM_ was homogenous across the brain. The three-compartment model yielded similar Akaike Information Criterion values to the two-compartment model, but allows more insight into physiology and is thus preferred. Overall, the protocol allowed simultaneous characterization of BBB and BCSFB dynamics, with the three-compartment model offering the most informative representation of water exchange.

## Introduction

The blood–brain barrier (BBB) and blood–cerebrospinal fluid barrier (BCSFB) are vital structures of the human brain which protect neuronal tissue from harmful pathogens, regulate the transport of metabolic, immunologic, and hormonal molecules between fluids and tissues to preserve homeostasis, and facilitate the clearance of waste products.^1–4^ The BBB refers to the set of membranes and junctions which form the boundary between blood and tissue found throughout the brain, while the BCSFB refers to the boundary between blood and CSF. The main exchange between blood and CSF is traditionally understood to occur at the choroid plexus (CP), a small structure located within the ventricles. The BCSFB is also implicated in the process of brain waste clearance by regulating the exchange of water and waste products between the blood and CSF. Disruptions in the function of the BBB and BCSFB are involved in a wide variety of neurological disorders such as Alzheimer’s disease, stroke, small-vessel disease, hydrocephalus, as well as the brain’s aging process.^4–12^ Therefore, assessing the function of the BBB and BCSFB provides crucial information about brain health and progression of brain diseases and preferably one would like to assess both barriers at the same time, because of the close interactions between blood water, CSF, and interstitial fluid (ISF).

Traditionally, MRI measurements of BBB and BCSFB leakage are performed using gadolinium-based contrast agents (Gd), however there are limitations to these techniques. Firstly, they are invasive, measuring the leakage rate of intravenously injected Gd across the BBB^13^ or the perivascular influx of Gd after intrathecal injection.^14,15^ For the latter, injections directly into the CSF may also disturb the equilibrium of the system we aim to characterize.^16^ Secondly, Gd crosses the barriers at a very slow rate unless there is substantial disruption to the tight junctions of the endothelial cells at the BBB and the epithelial cells at the BCSFB, which regulate the paracellular movement of Gd.^17,18^ Whilst Gd has been found to cross the barriers at measurable levels in several neurodegenerative diseases as well as in the aging brain,^13,19–25^ using a smaller tracer may offer improved sensitivity to subtle or early-stage barrier impairment. Additionally, contrast-enhanced techniques cannot detect damage to other transport routes across the barrier, as Gd exclusively traverses via paracellular pathways. There is also a growing concern with the deposition of Gd in the brain over time from repeated contrast injections. Therefore, there has been increasing interest in using water as an endogenous contrast medium to measure BBB and BCSFB function as it is fully non-invasive, has the potential to provide measurements of more subtle disruptions in function, and represents healthy physiology, that is, existing processes of exchange which are naturally present in the brain.

Most of the proposed methods to assess water transport across the BBB and BCSFB employ arterial spin labeling (ASL) MRI,^26–31^ a technique which is generally used to measure cerebral perfusion. By optimizing ASL labeling parameters and readout strategies, blood water can be tracked as it traverses from large vessels into the tissue or CSF. Because of the different nature of the tissues and fluids involved, different techniques are used for the assessment of the integrity of the BBB and BCSFB. In all cases, multiple labeling durations (LD) and/or post-labeling delays (PLD) are employed to capture the dynamic process and either multiple echo times (TE) or different levels of flow-crushing are performed to determine in what compartment the label resides. When exploiting the difference in T_2_ between compartments, the technique of choice to characterize the BBB is the combination of time-encoding with a T_2_-preparation (T_2prep_) module.^29,32–34^ Time-encoding (or Hadamard encoding) is an efficient method for acquiring multiple LD/PLDs, especially short ones like those required for BBB imaging (~200–2000 ms). T_2prep_ offers the advantage of allowing flexible effective echo times (eTEs), however it considerably increases scan time as only one eTE can be acquired per TR. For BBB water transport measurements, conventionally four short (<200 ms) eTEs are acquired. For BCSFB water exchange, we have previously proposed to use separate scans with different LD/PLDs in combination with a multi-TE gradient and spin-echo (GRASE) readout.^28^ For this method, longer LD/PLDs (~500–4000 ms) are acquired as water exchange from blood to CSF is believed to occur more slowly and to a lesser extent than exchange from blood to GM.^28^ Consequently, Hadamard encoding was found not very efficient and did not significantly reduce scan time. To isolate water signal in the CSF, extra-long TEs (>500 ms) must be acquired as CSF has a very long T_2_, which makes multi-TE GRASE an effective readout option. It has the advantage of acquiring multiple TEs simultaneously in one TR, albeit with longer inter-echo spacing. These long TE spacings (~260 ms in our previous work) do not allow proper separation of blood and GM signal because of their relatively short and similar T_2_s. In this work, we aim to reconcile the advantages and disadvantages of these two techniques (see Table 1) to measure blood water exchange to both GM and CSF in a single scan. In order for our protocol to contain a wide range of both TEs and PLDs, we combined T_2prep_ and a multi-echo GRASE readout into one sequence and sequentially acquired multiple LD/PLDs.

**Table 1.** Characteristics of the four compartmental models used in this work.

Model	Compartments (T_2_ (ms))	TEs	Fitted parameters	Schematic
*2-comp all*	1. Blood + GM (100) 2. CSF (1500)	All	CBF, ATT, *K_bl_*_ → csf_	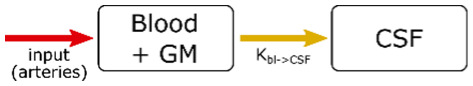
*3-comp all*	1. Blood (200) 2. GM (70) 3. CSF (1500)	All	CBF, ATT, *K_bl_*_ → gm_, *K_bl_*_ → CSF_	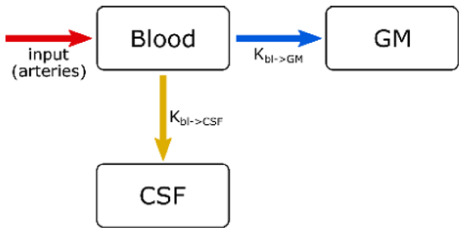
*2-comp* BBB	1. Blood (200) 2. GM (70)	1–4	CBF, ATT, *K_bl_*_ → gm_	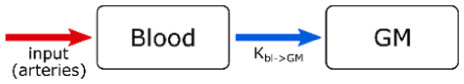
*2-comp* BCSFB	1. Blood + GM (100) 2. CSF (1500)	1, 5, 9, 13	CBF, ATT, *K_bl_*_ → CSF_	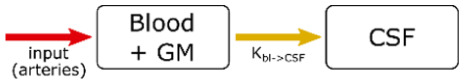

This study’s aims are therefore twofold:

• To investigate the use of a combination of T_2prep_ and multi-TE acquisition for measurement of ASL signal at both intermediate and long TEs.
• To compare the use of two- and three- compartment models tailored to differentiate the contributions from several compartments (blood, GM, or CSF) to the ASL-signal for characterization of water transport both from blood-to-GM (BBB) as well as from blood-to-CSF (BCSFB).

## Methods

### MRI acquisition

**Figure 1.**
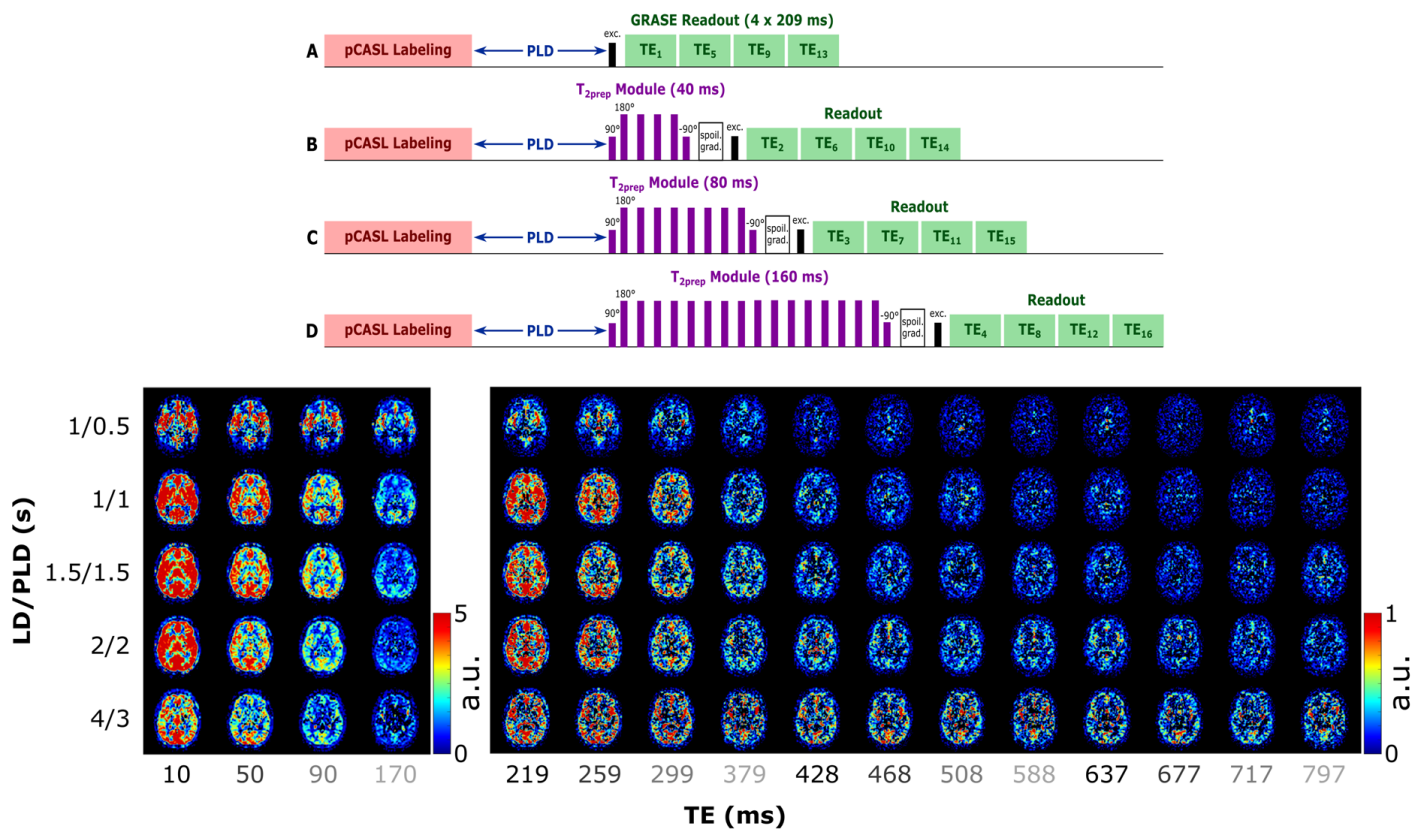
(Above) Schematic representation of the PCASL sequence used in this work. The image is not to scale (e.g. the length of the echoes in the multi-TE readout is longer than the longest T_2prep_ module). Each of the T_2prep_ module lengths (A–D) is acquired in a separate dynamic, resulting in an interleaved echo acquisition. (Below) ASL signal in a single slice for all LD/PLDs and TEs. Different scales allow the contrast to be preserved in the longer TEs. Echo times that are acquired sequentially within one readout are shown with the same grayscale intensity to highlight the interleaved acquisition.

Data was acquired from six healthy volunteers (1 M/5 F, age 21–63) on a Philips Achieva XT 3 T system (Philips, Best, the Netherlands) with a 32-channel head coil. This study was approved by the Medisch Etische Toetsingscommissie (METC), Leiden–Den Haag–Delft. The main imaging sequence in this protocol is a pseudo-continuous ASL (PCASL) scan with multi-echo 3D GRASE readout (as described in our previous work^28^), with the addition of a T_2prep_ module before readout. Here, the multi-TE readout has been shortened to four TEs acquired sequentially (TE = 10 + *n* × 209 ms, *n* = 0:3). The TSE factor was 80, the slice oversampling factor 1.8, and the in-plane acquisition matrix was 64 × 60 (RL × AP), reconstructed to a volume of 80 × 80 × 28 voxels of 3 × 3 × 6 mm^3^. The EPI readout in the phase-encoding direction (AP) was divided into four segments (15 lines/segment). A T_2prep_ module was incorporated to increase the sampling density of the T_2_ decay curve, allowing more effective separation of tissue signals—particularly blood and GM, which have rather similar T_2_ values and therefore require closer effective echo times to be distinguished. Four T_2prep_ modules were applied with lengths of 0 (no T_2prep_), 40, 80, and 160 ms. These were cycled along the dynamics, that is, the acquisition of echoes was interleaved: in the first dynamic (eTE = 0), TEs = 10 + *n* × 209 ms, *n* = 0:3 (i.e. TEs #1,5,9,13) were acquired, in the second dynamic (eTE = 40), TEs = 50 + *n* × 209 ms, *n* = 0:3 (TEs #2,6,10,14), and so on, for a total of 16 TEs (10, 50, 90, 170, 219, 259, 299, 379, 428, 468, 508, 588, 637, 677, 717, and 797 ms). This represents a good compromise between short-TE and long-TE acquisitions to allow distinction between the three main compartments of interest (blood, GM and CSF). In order to keep the effective length of the PLD the same between different T_2prep_s, the module was inserted after the PLD (see Figure 1). Two background suppression (BGS) pulses were applied and their timings were optimized to minimize background signal from GM, white matter (WM), and CSF at the end of the PLD and before the T_2prep_ module. The sequence was repeated five times with variable LD/PLDs (1/0.5, 1/1, 1.5/1.5, 2/2, 4/3 s) to cover a range of timings from the arrival of blood in the voxel to the transport of water into the CSF. Repetition times (TR) were minimized for the different durations of the ASL preparation (TR = 3000, 3500, 4500, 5500, 8500 ms, respectively). Finally, an M0 scan was acquired with the same imaging parameters as the PCASL scans, but without labeling. This scan included a single TE of 10 ms and a TR of 10,000 ms (to ensure the full recovery of the GM signal). The length of individual PCASL scans varied from 6:42 to 19:00 min and the total length of this protocol was ~1 h. To avoid order-effects, we randomized the order of the PCASL scans in each participant.

This research was carried out in accordance with our institution’s IRB regulations (Medisch Etische Toetsingscommissie (METC), Leiden–Den Haag–Delft) and the Declaration of Helsinki.

### Analysis pipeline

ASL control and label images were pair-wise subtracted to calculate ASL signal at all LD/PLDs and TEs. A CSF mask was created by thresholding the control image at the longest TE, and a GM mask was obtained by thresholding the ASL signal of the LD/PLD 2/2 s image (the closest to typical ASL perfusion parameters where there is maximal contrast between the GM and other tissues and fluids). The thresholds for these masks were adjusted for each subject by an experienced observer (first author who had 4 years of experience with ASL-data) to retain as much as possible the anatomical features such as the cortex gyri and CSF-filled sulci. The WM mask was isolated by removing the GM and CSF mask voxels from a whole brain mask. Finally, a CP mask was obtained by isolating the posterior part of the ventricles where the CP is predominantly located and using the same threshold as the GM mask to exclude the CSF surrounding the CP.

Two ROIs were created by superimposing an elliptical shape onto (1) a single slice in the middle of the brain (slice 15), comprising the majority of the left MCA territory and avoiding the ventricles (left MCA ROI) and (2) in a high slice of the brain (slice 22), comprising most of the left hemisphere in that slice (high slice ROI). The ASL signals from these ROIs were averaged, combining signal from all tissues and fluids within the elliptical regions, and four multi-compartmental models, detailed in the following section, were fitted to the resulting data. In addition, voxelwise fitting of these models was performed to generate whole-brain maps of the corresponding model parameters. Prior to fitting, the ASL signal was smoothed with a gaussian kernel (3 × 3 voxels, σ = 2 voxels) to reduce noise. To enable comparison of parameter values across brain regions, the estimated parameters were averaged within the following tissue region ROIs: GM (GM mask slice 15 and up), subarachnoid space (SAS; CSF mask slice 15 and up, excluding the ventricles), CP (CP mask), and WM (WM mask, used as a control region). Because of the small number of subjects (i.e. low statistical power), the parameters were compared qualitatively instead of performing formal statistical analyses.

### Compartmental models

Four models were used to characterize the exchange of water between the blood, GM, and CSF compartments in this study. Detailed equations for the models can be found in the Supplementary Material. In brief, the first model (*2-comp all*) is comprised of two compartments, where the blood and GM are combined into one compartment with an intermediate T_2_, and the CSF is the other. This is the same model that was used in our previous work on BCSFB characterization,^28^ and it was chosen at the time because of the difficulty in separating the blood and GM into distinct signals because of their short and similar TEs (~200 and 70 ms, respectively) compared to the echo time spacing of ~260 ms in that study. Here, we have shorter inter-echo spacing, and the restriction to two compartments should prove unnecessary (this model was however included here for comparison to our previous data). The second model (*3-comp all*) is a three-compartment model with blood, GM, and CSF in separate compartments. The third and fourth models attempt to single out water exchange across the BBB and BCSFB, respectively, by fitting only a subset of the TEs. These resemble acquisitions focused on measuring either BBB or BCSFB measurements and serve as a reference against which to compare the results of our combined sequence. The third model (*2-comp BBB*) is a two-compartment model with the blood and GM as compartments, ignoring the CSF signal. This model only uses the first four echo times (i.e. first GRASE echo for the different T_2prep_ modules) since blood and GM signal mostly contributes to the signal of the shorter TEs. The fourth model (*2-comp BCSFB*) is a two-compartment model, with the same compartments and equations as the first model. The difference with this model is that it only includes the four GRASE TEs of the first dynamic (i.e. the four TEs acquired sequentially within one readout without a preceding T_2prep_ module). These last two models not only allowed us to test the use of fewer TEs for BBB and BCSFB quantification, but also focus on a single method for acquisition of multiple TEs (T_2prep_ and multi-TE readout, respectively), so that any errors resulting from the combination of the two are avoided. Table 2 summarizes the characteristics of the four models: the assumed T_2_s^35–37^ of the compartments, which TEs are used, a list of the fitted parameters, and a schematic representation of the exchange mechanics. We modeled only the unidirectional exchange of water from the blood towards other compartments, as any ASL-signal originating from water that has exchanged back into the blood should be negligible (both because it represents a very small fraction of signal, and because it would necessitate longer LD/PLD). For all models, the T_1_s of blood, GM, and CSF are assumed to be 1650,^38^ 1200,^37^ and 4300^39^ ms, respectively, and the inversion efficiency (α) is set equal to 0.85 (combination of the labeling efficiency^40^ and the BGS pulses inversion efficiency^41^). A single average M0 value was calculated for each subject by averaging the signal of the M0 scan over the GM mask and dividing it by the blood–brain partition coefficient, λ = 0.98 ml/g.^42^

**Table 2.** The median AICc values in the analyzed ROIs for the *2-comp all* and *3-comp all* models.

Subject	Whole brain			Gray matter ROI			White matter ROI			Subarachnoid space ROI			Choroid plexus ROI		
	*2-comp*	*3-comp*	Difference	*2-comp*	*3-comp*	Difference	*2-comp*	*3-comp*	Difference	*2-comp*	*3-comp*	Difference	*2-comp*	*3-comp*	Difference
Subject 1	1667.05	1669.23	2.18	1795.90	1798.10	2.20	1706.25	1708.45	2.20	1779.53	1781.73	2.20	1720.82	1723.28	2.46
Subject 2	1704.70	1706.85	2.15	1838.27	1840.47	2.12	1758.39	1760.56	2.17	1796.86	1795.03	2.17	1777.83	1779.90	2.07
Subject 3	1632.89	1635.07	2.18	1747.82	1749.99	2.17	1653.41	1655.62	2.21	1714.15	1716.38	2.23	1721.66	1723.76	2.10
Subject 4	1690.58	1692.72	2.14	1809.36	1811.62	2.26	1711.35	1713.55	2.20	1777.76	1779.85	2.09	1702.02	1704.22	2.22
Subject 5	1794.94	1797.11	2.17	1935.74	1937.95	2.21	1847.28	1849.45	2.17	1905.22	1907.50	2.28	1857.39	1859.55	2.16
Subject 6	1735.55	1737.71	2.16	1838.18	1840.38	2.20	1779.49	1781.73	2.24	1813.12	1815.33	2.21	1801.10	1803.31	2.21

### Akaike Information Criterion (AIC) analysis

The *2-comp all* and *3-comp all* model fits were compared using the small sample size corrected Akaike Information Criterion (AIC), calculated as^43–45^:

$$AICc=2k_{m}-2\left(MLL\right)+\frac{2k_{m}\left(k_{m}+1\right)}{n-k_{m}-1}$$

where *n* is the number of data points (*n_3-comp all_* = *n_2-comp all_* = 5 PLDs × 16 TEs = 80), *k_m_* is the number of estimated model parameters (for the *2-comp all* model these are CBF, ATT, *K_bl_*_ → CSF_ so *k_m_*_,*2-comp all*_ = 3, for the *3-comp all* model *K_bl_*_ → GM_ is also included so *k_m_*_,*3-comp all*_ = 4) and MLL is the maximum value of the log-likelihood for the model given by:

$$MLL=-\frac{n}{2}\ln\left(\frac{RSS}{n}\right)$$

where RSS is the residual sum of squares. Voxelwise AIC values were evaluated across the whole brain, GM, CP, SAS, and WM ROIs.

## Results

### ASL signal

The measured ASL signal (Figure 1) is consistent with literature and previous work (note that two different scales are used for the first four echoes and the rest, to visualize contrast at long TEs where the signal is low). When focusing on the first TE (first column) we see the early arrival of blood in the vasculature in the first time point, then the signal appears in the GM as a perfusion pattern (LD/PLD 1/1–2/2 s) before fading away. In longer TEs (>500 ms), the CSF signal becomes apparent, with a slower inflow (it appears mostly from LD/PLD 2/2 s and longer) and slow decay through the TEs. The evolution of the signal through the TEs shows rapid decrease for blood and GM signals and slower decay in the CSF (CP and SAS). As seen in our previous work,^28^ CSF signal from blood-to-CSF water exchange is present throughout the brain, and not exclusively in the choroid plexus.

### Signal fractions

**Figure 2.**
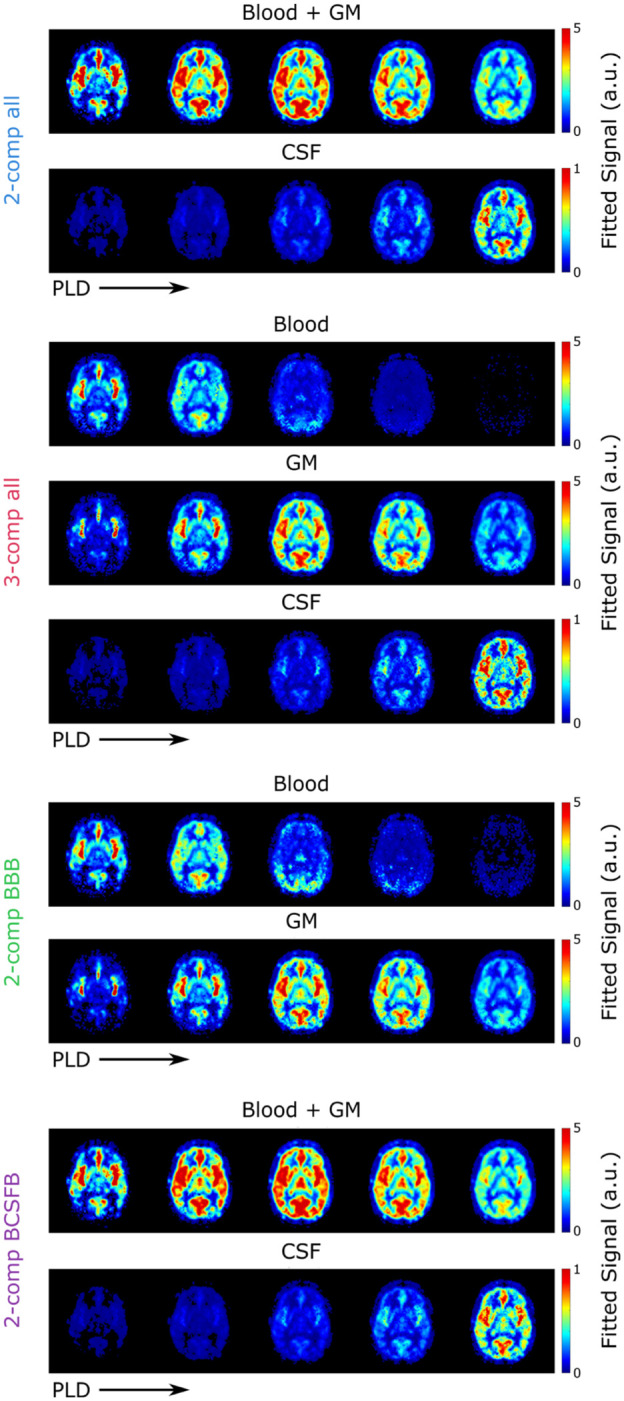
Relative amount of signal present in each compartment at TE = 10 ms and all LD/PLDs used in this experiment as fitted with the four models.

Using the parameter maps estimated from all compartmental models, the amount of signal arising from each compartment can be calculated from the model equations. These signal fractions are shown in Figure 2 for a single slice of subject 5 for all LD/PLD pairs of this study. Results of the *2-comp all* and *2-comp BCSFB* models are similar, and comparable to the results of our previous work using this same model. The *2-comp all*, *3-comp all*, and *2-comp BCSFB* models result in comparable CSF signals in terms of timing, location, and intensity, with the *3-comp all* model showing the highest CSF signal.

In our previous work, the blood and GM compartments were combined into a single compartment, because the large inter-echo spacing of the data did not allow their short T_2_s to be distinguished and hence resulted in a large amount of overlap between their signal maps when using a three-compartment model. Here, in the *3-comp all* model maps, there is some residual overlap between the two compartments, but the resulting maps show distinct patterns for blood and GM, for example, there is very little signal in the GM in the first time point, by the second time point the blood signal has completely disappeared from the large arteries, and the signal decay through time is much faster in blood than GM. However, in the second time point, the GM signal appears paradoxically more concentrated around blood vessels and the blood signal is more spread out. The overlap between blood and GM is greater in the *2-comp BBB* model than the *3-comp all* model.

Supplementary Figure S1 presents residual maps illustrating the differences between the total fitted signal and the measured signal at each TE/PLD for both the *2-comp all* and *3-comp all* models, for all subjects. Both models exhibit low residuals at longer TEs but show higher residuals at shorter TEs. This is expected, as early TEs capture signal contributions from all compartments, while at later TEs, the CSF signal becomes more isolated, as signals from blood and GM have already undergone significant T_2_ decay. Variations across PLDs are also evident: the *3-comp all* model tends to overestimate total signal at shorter PLDs and underestimate at longer PLDs, whereas the *2-comp all* model generally overestimates at longer PLDs. These trends were consistent across different imaging slices.

Additionally, Supplementary Figure S2 shows a video of the signal obtained from the *3-comp all* model for an artificial bolus of LD = 3 s for PLDs varying from 0 to 5 s. This shows a more intuitive view of the exchange of water between the three compartments, with a single (virtual) LD and therefore a smoother transition in time.

### AIC results

As shown in Table 2 and Supplementary Figure S3, the AIC values for the *2-comp all* model are consistently lower across all regions than the *3-comp all* model. However, the differences in their AIC values are minimal, with the largest observed difference in median AIC being 2.46.

### ROI analyses: Left MCA and high slice ROIs

**Figure 3.**
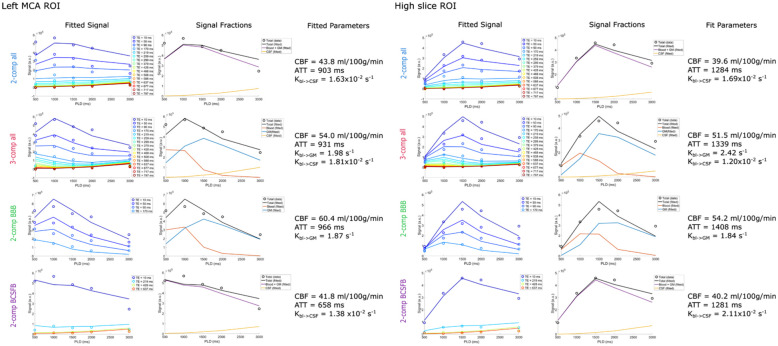
Model fitting of the signal in the left MCA territory ROI (single slice, containing 390 voxels) and the high slice ROI (single slice, containing 338 voxels) of subject 5 for all four models. The first column shows the signal (circles) and fitted curves (lines) for the different echo times. The second column shows the signal in each compartment based on the fit, and the third column shows the fitted parameters.

Figure 3 shows the fit of the four different models for the left MCA ROI and high slice ROI. While both ROIs combine a mixture of signal from blood, GM, and CSF, because of the geometry of acquisition the high slice ROI contains more SAS CSF than the MCA ROI. Based on the GM, WM, and CSF masks, 45% of the voxels in the left MCA ROI contained GM, 37% contained WM, and 22% CSF. For the high slice ROI, the proportions shift to 36% GM, 46% WM, and 27% CSF. Looking at the signal curves, we see earlier arrival of the signal in the MCA territory and generally higher signal intensity. In the MCA ROI, the CBF is higher, the ATT shorter, *K_bl_*_ → GM_ larger and *K_bl_*_ → CSF_ smaller, consistent with the closer proximity to the circle of Willis and higher fraction of GM. In both ROIs, by visual inspection of the fitted curves the first model (*2-comp all*) shows lower correspondence with the data, especially for the shorter echo times (50–170 ms), than the *3-comp all* model which shows a better fit at these echo times. Compared to the *3-comp-all* model, *2-comp BBB* overestimates CBF, and *2-comp all*/*2-comp BCSFB* underestimate it, and the same is true for ATT. Differences between signal fractions are subtle, but we can see when comparing the *3-comp all* model to *2-comp BBB* that in the latter case, the CSF signal appears to be absorbed into both the GM and blood compartments as both show increased signal. In the case of the *2-comp all* and *2-comp BCSFB* models, some of the CSF signal appears to be absorbed into the blood + GM compartment as the fraction of CSF-signal here is lower than in the *3-comp all*. From visual assessment of the fits, the *2-comp BBB* model performs better in the MCA territory ROI than in the high slice ROI, because of the higher GM proportion here. On the contrary, the *2-comp BCSFB* performs better in the high slice ROI, due to a greater percentage of CSF in this ROI.

### Parameter mapping: Whole brain, GM, WM, and CP ROIs

**Figure 4.**
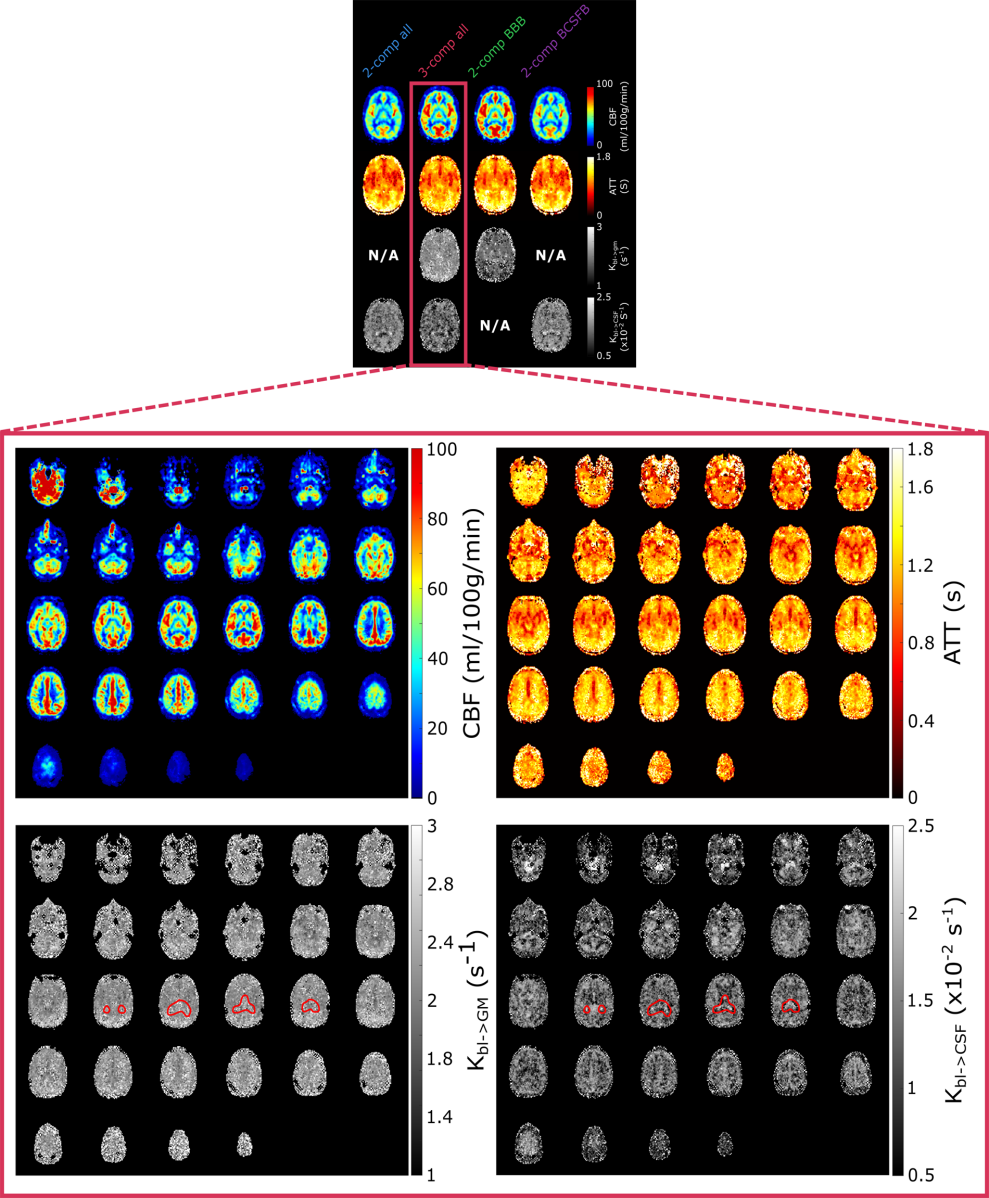
(Above) Single slice parameter maps for all models in subject 5. (Below) Whole-brain parameter maps in subject 5 for the *3-comp all* model. The choroid plexus is circled in red. The first two and lowest slices of the brain (top left corner) intersect with the labeling plane, explaining the presence of high erroneous signal.

Parameter maps for a single slice created by all models (subject 5) are shown in Figure 4 (for full brain maps of two other subjects see Supplementary Figures S4 and S5). The CBF and ATT maps are consistent with literature. The CBF shows a typical perfusion pattern, while the ATT map highlights large arteries (the middle cerebral arteries and the anterior cerebral artery) and prolonged ATT in the posterior region of the brain and border zones. *K_bl_*_ → GM_ is fairly uniform across the brain with notably lower values for the *2-comp BBB* model. Finally, *K_bl_*_ → CSF_ maps show contrast between the CP (situated at the center of this slice), with visibly higher values (because of the presence of the BCSFB) than the rest of the brain. Exchange rates are otherwise more or less homogenous across the GM, with more variations seen in the *3-comp all* model, where regions with limited *K_bl_*_ → CSF_ exchange appear especially in the WM and *K_bl_*_ → GM_ is more homogenous throughout the brain.

Whole-brain parameter maps are shown in Figure 4 for the *3-comp all* model. Here the CP is encircled in red to highlight differences in *K_bl_*_ → GM_ and *K_bl_*_ → CSF_ in this region. The contrast between brain regions is more visible in the whole-brain maps and we can see how parameters vary across the entire brain. The CP in particular is clearly visible in the *K_bl_*_ → CSF_ map with its elevated exchange values. There is also contrast between the gray and white matter, with slower exchange in the WM, and faster *K_bl_*_ → CSF_ exchange in the higher slices where cortical GM and SAS are bordering. The *K_bl_*_ → GM_ map shows less contrast, but appears to result in lower values around the large arteries (circle of Willis and ACA).

**Figure 5.**
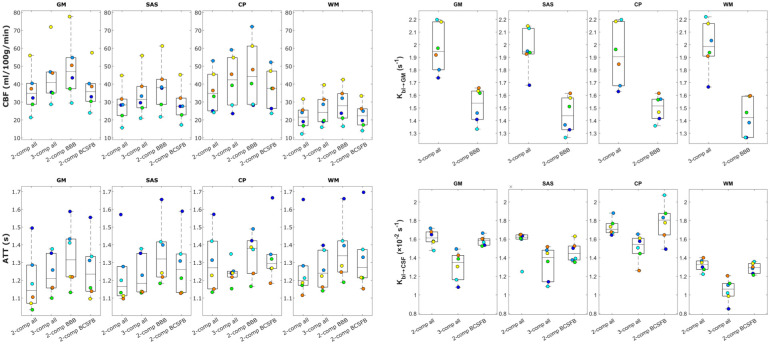
Average values of model parameters fitted with the four compartmental models for all subjects in GM, SAS, CP, and WM ROIs. Note that *K_bl_*_ → GM_ is denoted referencing only GM rather than other extravascular tissue compartments that label may have exchanged into (such as WM) because during model fitting the assumed extravascular T_2_ is that of GM.

For a direct spatial comparison of these parameters across different tissue regions, the maps were averaged over the GM, SAS, CP, and WM ROIs. The averaged parameter values are shown in Figure 5 for all subjects and models. It is important to note that, although these ROIs were defined using tissue-specific masks and primarily represent regions dominated by the corresponding tissue types, they were not assumed to consist exclusively of a single tissue type. As a result, the ROIs are likely to include partial-volume contributions from surrounding tissues. For example, voxels within the GM ROI may contain partial-volume contributions from the SAS, such that at late TEs CSF signal is present within these voxels, allowing quantification of *K_bl_*_ → CSF_ reflecting label exchange into this CSF compartment. CBF values are fairly consistent with literature, with the exception of the average GM CBF between 30 and 50 ml/100 g/min, which is lower than average.^46,47^ The values in the CP around 35 ml/100 g/min^48^ and in the WM around 20 ml/100 g/min^47^ however are appropriate. For the ATT, values are also within the expected range, with longer transit times in the CP and WM as commonly observed in the literature. The tendency previously seen in ROI analyses and maps for the *2-comp BBB* model to overestimate both CBF and ATT and for the *2-comp all* and *2-comp BCSFB* models to underestimate them compared to the *3-comp all* model is clearly visible in these graphs. For *K_bl_*_ → GM_, differences between the models are far greater than differences between brain regions (~2 s^–1^ for all regions using the *3-comp all* model, ~1.4 s^–1^ for the *2-comp BBB*). Potential reasons for the low contrast in this parameter between brain regions will be addressed in the discussion. Finally, *K_bl_*_ → CSF_ is approximately a factor 100 lower than *K_bl_*_ → GM_, with values ranging from ~1–1.4 × 10^−2^ s^–1^ in the WM ROI, to ~1.2–1.6 × 10^−2 s^–1^^ in the GM and SAS ROIs, to ~1.5–2 × 10^−2^ s^–1^ in the CP. Here the contrast between brain regions is clear; there is fastest exchange in the CP, and the slowest exchange is seen in the WM.

## Discussion

In this work, we aimed to simultaneously measure water exchange in the human brain from the blood to the GM and to the CSF. We also compared the CBF, ATT, and exchange parameters *K_bl_*_ → GM_ and *K_bl_*_ → CSF_ obtained by fitting four different compartmental models to our data. Our key takeaways are that our imaging protocol allows the measurement of ASL signal in the blood, GM, and CSF with its combination of LD/PLDs and echo times, and additionally that all compartmental models demonstrated comparable applicability to the data. Therefore, including the second exchange parameter in the *3-comp all* model did not meaningfully improve or degrade the quality of the fit. Nevertheless, we believe that the *3-comp all* model most accurately represents the underlying physiology since it provides information about the separate water exchange processes between the three tissue compartments where exchange is thought to occur during our measurements.

The combination of T_2prep_ and multi-echo readout was effective in measuring ASL signal for a large range of TEs with sufficient small inter-echo spacing. The ASL signal (Figure 1) is consistent with literature, and shows a relatively smooth transition from high signal in the short TEs to low signal in long TEs. The longest TEs (from ~500 ms) effectively isolate CSF signal as seen in the complete decay of blood and GM signal in the first two time points at longer TEs, followed by the gradual increase in long-TE signal consistent with the later arrival of labeled water in the CSF.

Furthermore, the proposed compartmental models provided good fits to the signal, with the *3-comp all* model resulting in the most informative representation. Analyses of the left MCA and high slice ROIs (Figure 3) show this fit in detail for all TE and PLD data points. All models offered reasonable fits to the data, and resulted in distinct parameter values for these two ROIs. These differences reflect variations in compartment dynamics consistent with the differing tissue compositions of the ROIs (see Section 3.2 for details on the proportions of GM, WM, and CSF in each ROI). For example, the high slice was associated with longer ATTs, higher blood-to-CSF water exchange rate (because of the presence of more CSF) and lower blood-to-GM exchange. By visual inspection, the *3-comp all* model showed the closest fit to the ROI data.

Turning to the brain-wide tissue region ROI analyses, the parameter maps shown in Figure 4 and the regional averages in Figure 5 demonstrate that the *3-comp all* model provided good contrast in *K_bl_*_ → CSF_ across brain regions, with faster exchange in the CP compared with the SAS, GM, and WM ROIs. This is consistent with the BCSFB at the CP being a known site of substantial water exchange.^49,50^ The similar *K_bl_*_ → CSF_ values observed in the SAS and GM ROIs indicate a more brain-wide phenomenon of water exchange from blood to CSF, in line with previous ASL studies.^28^ The *K_bl_*_ → CSF_ measurements in the GM ROI may be interpreted as reflecting label exchange from blood into CSF compartments (e.g. periarterial or subarachnoid spaces), or label that first enters parenchymal GM compartments and subsequently moves into CSF, for example, via periarterial or perivenous spaces. With respect to this latter pathway, it seems unlikely that ASL label exists long enough to fully resolve a blood → GM → CSF transport route. A rough estimate of the time required for a spin to freely diffuse from a penetrating arteriole to a perivascular space via the brain tissue—assuming a random walk distance of ~280 µm and a diffusion coefficient of D = 1.0 × 10^−3^ mm^2^ s^–1^ yields an approximate diffusion time of 13 s.^51–53^ This timescale exceeds that of our ASL measurements, even for the longest LD/PLD pair (4/3 s). In addition, we expect T_1_ decay of the label to be too rapid for any detectable signal to survive this pathway as label would experience the tissue T_1_ for considerable time. considerable time. Consequently, we believe that the measured signal most likely reflects direct blood-to-CSF exchange, and the *3-comp all* model is defined to represent this more probable route. This is, however, speculative and further studies would be valuable to determine whether a GM-mediated pathway could occur within the timescales of these measurements. Results for the *K_bl_*_ → GM_ parameter, are more intuitive to interpret. The values for this parameter were more homogenous throughout the brain, and differences between averages in the four regions of the brain were negligible compared to the difference between the results of the *3-comp all* and *2-comp BBB* models. The *2-comp BBB* model consistently yielded slower exchange (lower *K_bl_*_ → GM_) than the *3-comp all* model, resulting in a higher fraction of signal in the blood compared to the *3-comp all* model (see Figure 2).

Several studies using multi-echo and diffusion-prepared ASL report *K_bl_*_ → GM_ values that closely match our *3-comp all* model estimates (1.6–2.3 s^–1^).^1,31,32,54–56^ Other studies report higher values (3.2–5 s^–1^),^55,57–59^ but these still align more closely with the *3-comp all* model than with the *2-comp BBB* model. A contrast-enhanced ASL study^60^ likewise shows good agreement with the *3-comp all* results. However, direct comparison across studies should be interpreted with caution. Aging effects,^55,57,58^ differences in acquisition sequences, and variations in modeling and fitting procedures all introduce substantial variability, limiting the reliability of cross-study comparisons. In contrast, literature on *K_bl_*_ → CSF_ is limited. The values of *K_bl_*_ → CSF_ obtained here are consistent with those from our previous multi-echo study,^28^ agreeing more closely with the *2-comp all* model estimates which is expected given the previous work applied a two-compartment model. Other techniques applied in humans include REXI,^61^ which has reported substantially higher exchange rate in the CP, though direct comparison is inappropriate due to differing acquisition and modeling assumptions. Taso and Alsop’s T_2_-prepared FLAIR approach^62^ indicates increased exchange in the CP, consistent with our findings, but does not yield a parameter directly comparable to *K_bl_*_ → CSF_. Several additional techniques have been proposed in animal models^63–65^ but have not yet been translated to humans. A major limitation of the field—and of the present work—remains the lack of validated human reference standards for CSF water-exchange measurements.

In addition, variability in T_2_ of arterial blood due to its dependency on oxygenation might have influenced the results.^66^ The similarity in values between brain regions could likewise be due to limitations of the fitting technique, or could indicate that the phenomenon which we aim to characterize, that is, the exchange of water from blood to tissue, is present throughout the brain at a comparable rate. The models employ literature values of GM for all brain tissues, that is, also for WM. This can be justified by their relative similar T_2_s compared to the much more different properties of CSF. Additionally, the *3-comp all* model not only resulted in acceptable parameter values and good contrast between brain regions, but it also provided better separation of the signal originating from the three compartments (Figure 2), especially with less overlap between blood and GM compartments compared to the *2-comp BBB* model. The remaining overlap between blood and GM could be due to the aforementioned variable blood T_2_, or we may simply be observing the blood-to-GM water exchange occurring closer to the large vessels in early time points leading to the appearance of vascular signal in the GM compartment in the first two PLDs. In addition, the compartmental models used do not include a macrovascular compartment (i.e. blood contained in the large arteries instead of arterioles and capillaries), and may thus break down in the presence of large arteries. Finally, the addition of the third compartment might be considered more realistic on theoretical grounds, as blood, GM, and CSF are three distinct compartments with water exchange from the blood to the latter two occurring at appreciably different rates and governed by barriers with unique cellular components and transport mechanisms.

The AIC analysis didn’t favor a specific model as both models yielded similar AIC values across all brain regions, with the *2-comp all* model exhibiting slightly lower AIC values. According to the literature, a difference in AIC (relative to the model with the lowest AIC value) in the range of 2–7 indicates a reasonable fit quality and thus that a model should not be disregarded.^67^ On average, the AIC difference between the *2-comp all* and *3-comp all* models does not exceed 2, suggesting that both models can be considered as providing comparable quality fits to the data. This finding indicates that the additional fitting parameter (*K_bl_*_ → GM_) in the *3-comp all* model does not lead to overfitting, a conclusion further supported by visual inspection of the fitted curves in Figure 3. Since both models demonstrate acceptable fit quality, we argue that for a similar level of error the *3-*comp *all* model is superior because it more accurately represents the underlying physiology. Specifically, it distinguishes between blood and GM as distinct compartments, allowing the two exchange parameters (*K_bl_*_ → GM_ and *K_bl_*_ → CSF_) to be extracted from a single sequence and fitting procedure, thus offering a more comprehensive view of brain water exchange dynamics.

There are a number of limitations to this study which should be noted. Firstly, the imaging sequences used are long, and a lot of optimization was needed to reach a satisfactory range of echo times and LD/PLDs which would fit into an hour of scanning. The timing was so limiting that the T_1_w image that would usually be acquired with such a protocol had to be forgone. A T_1_w scan is typically used to create GM, WM, and CSF masks which can subsequently be coregistered to the ASL data, but here we had to use more indirect masking techniques which may have resulted in less effective separation of tissue types. This might also explain the fact that the GM CBF values were lower in this study than typically observed in literature. The long scan times limit the clinical translatability of this method for which it may be more efficient to employ Hadamard time-encoding for BBB characterization, along with separate longer LD/PLD and TE T_2_-prepared acquisitions as implemented in this paper for the BCSFB. Reducing the number of sampled TEs and/or PLDs could also be beneficial however extensive simulations, such as leave-one-out cross-validation, would be required to ensure model sensitivity to exchange time and hemodynamic parameters is maintained.

Secondly, the ASL signal did show some instability through the TEs, with some small increases appearing at longer TEs. This is also observed when averaging signal over ROIs and plotting over TEs (shown in Supplementary Figure S6 for the two ROIs), where a slight saw-tooth effect was present. We however found that these variations were small in comparison to the natural signal decay along TEs, but these could still induce some errors in the analysis of this signal. This pattern could be caused simply by the interleaved nature of the acquisition of TEs with natural fluctuations in perfusion and physiological processes over the long scan time, or it could arise from an interaction between the T_2prep_ module and the readout module (and BGS). However, this behavior was different in different subjects (i.e. signal peaks did not occur at the same TEs). These variations were deemed acceptable in the context of this work, but further improvements in sequence design could alleviate this issue, for example, by limiting the overall temporal footprint.

Additionally, these data are of course acquired in a small cohort of healthy individuals, and it therefore did not have statistical power to study the relationship between water exchange rates and, for example, age and sex. Testing this technique in larger groups of subjects as well as in patient populations is critical to reach a better understanding of the role of brain water exchange in health, aging, and disease.

Finally, the most relevant limitation of this work is the absence of a reliable reference standard for validating water-exchange measurements. As a result, particularly for *K_bl_*_ → CSF_ which is a novel exchange parameter, some analyses remain qualitative or speculative. Validation will require comparison with techniques that probe the same mechanisms of water transport across the BBB and BCSFB. Existing MRI measures of BBB function, such as DCE-MRI, are not directly comparable to water-exchange metrics: gadolinium transport rate reflects function of different BBB mechanisms and studies measuring both modalities show that the two measures can be related but not in a one-to-one manner, with correlations present in some regions or cohorts but absent in others.^68–71^ Although H_2_^15^O PET uses water as a tracer, and is the reference method for in-vivo BBB water permeability, it lacks the ability of MRI to differentiate compartments on compartment specific parameters such as the T_2_ and also employs different kinetic modeling, making the measures non-equivalent.^72^ H_2_^17^O MRI^73^ is an avenue that shows promise in regard to BCSFB characterization, although its translation to the human brain may be difficult because of its prohibitive cost.

In conclusion, we find that the combination of a T_2prep_ module with a multi-echo 3D GRASE readout allows the non-invasive measurement of ASL signal at a wide range of echo times necessary for the simultaneous characterization of the BBB and the BCSFB. The proposed compartmental models were effective in measuring perfusion and exchange parameters, and in particular the three-compartment model showed promise to provide a more complete picture of the dynamics of water exchange between blood, GM, and CSF.

## Supplemental Material

sj-docx-1-jcb-10.1177_0271678X261429042 – Supplemental material for Simultaneous measurement of water transport across the blood–brain and blood–CSF barrier in the human brain with arterial spin labeling MRI Supplemental material, sj-docx-1-jcb-10.1177_0271678X261429042 for Simultaneous measurement of water transport across the blood–brain and blood–CSF barrier in the human brain with arterial spin labeling MRI by Léonie Petitclerc, Helena Durrant, Lydiane Hirschler, Lena Václavů and Matthias J.P. van Osch in Journal of Cerebral Blood Flow & Metabolism
