# Supplementary material for: Simultaneous measurement of water transport across the blood–brain and blood–CSF barrier in the human brain with arterial spin labeling MRI
Source: J Cereb Blood Flow Metab. 2026 Mar 17:0271678X261429042. Online ahead of print. doi: 10.1177/0271678X261429042 (PMC12999538; doi:10.1177/0271678X261429042)
Supplement: sj-docx-1-jcb-10.1177_0271678X261429042 – Supplemental material for Simultaneous measurement of water transport across the blood–brain and blood–CSF barrier in the human brain with arterial spin labeling MRI [file sj-docx-1-jcb-10.1177_0271678X261429042.docx]

**Model equations**

In this appendix the subscript x denotes any of the compartments (bl, GM, or CSF).

The following models were adapted from Gregori et al. ^32^ for PCASL and additional compartments.

**Two compartment model solution**

Equation 1:

$$t=LD+PLD$$

Equation 2:

$$R_{\left( 1,2 \right)X}=\frac{1}{T_{\left( 1,2 \right)X}}$$

Equation 3:

$$K_{bl\to(GM,CSF)}=\frac{1}{T_{bl\to(GM,CSF)}}$$

The equations for the two 2-compartment models (*2-comp BBB* or *2-comp BCSFB*) are equivalent. The equations for the *2-comp BBB* model are given below, but they take on the same form, after substituting the GM-labels with CSF and bl with bl+GM, when describing the *2-comp BCSFB* model. Therefore, the equations are only given once, with the bl/GM compartments, but they can easily be modified to represent the bl+GM/CSF compartments instead.

To calculate the expected signal at a certain TE (denoted S_x_), we must first calculate the signal S_1X_ before the excitation pulse. Before the labeled spins are excited into the transverse plane, the signal intensity is governed by longitudinal relaxation. Additionally, exchange of spins between compartments alters signal intensity in each compartment, leading to an apparent change in T_1_ relaxation:

Equation 4:

$${R'}_{1bl}= R_{1bl}+K_{bl\to GM}$$

Equation 5:

$${R'}_{1GM}= R_{1GM}+K_{bl\to GM}$$

Where R_1X_ is the T_1_ relaxation rate of a given compartment and K_bl🡪gm_ is the rate of transport of water from the blood compartment to the GM compartment. Although exchange of spins causes a slower apparent T_1_ decay in the GM compartment, we define ${R'}_{1GM}$ by adding $K_{bl\to GM}$ to the intrinsic relaxation rate; the expected behavior is then recovered by subtracting a term that accounts for exchange in Equation 7.

In their integral form, the equations are:

Equation 6:

$$S_{1bl}\left( t \right)=2M_{0}CBF\int_{0}^{t} c_{1}\left( t^{'} \right)r_{bl\to GM}\left( {t-t}^{'} \right)m_{1bl}\left( t-t^{'} \right)ⅆt^{'}$$

Equation 7 :

$$S_{1GM}\left( t \right)=2M_{0}CBF\int_{0}^{t} c_{1}\left( t^{'} \right)(1-r_{bl\to GM}\left( {t-t}^{'} \right))m_{1GM}\left( t-t^{'} \right)ⅆt'$$

With $c_{1}\left( t^{'} \right)$ the input function describing the arrival of water signal from the arteries into the imaging voxel, $r_{bl\to GM}(t)$, the residue function associated with the transport of water from the blood to the GM compartment, and $m_{(1bl,GM)}(t)$ the magnetization relaxation function which describes the loss of signal due to T_1_ relaxation.

Equation 8:

$$r_{bl\to GM}\left( t \right)= e^{-K_{bl\to GM}t}$$

Equation 9:

$$m_{1\left( bl,GM \right)}\left( t \right)= e^{-R_{1(bl,GM)}t}$$

Equation 10:

$$c_{1}\left( t \right)= \left\{ \begin{aligned} 0, t<\mathrm{ATT} \\ \alpha e^{-R_{1bl}ATT}, \mathrm{ATT}\leq t<LD+\mathrm{ATT} \\ 0, LD+\mathrm{ATT}\leq t \end{aligned} \right.$$

With the three parts of the input function representing

1. The time before the arrival of the labeled bolus in the imaging voxel

2. The time after the leading edge of the bolus has arrived and before the trailing edge arrival

3. The time after the bolus has completely entered the voxel

With the solution:

For t < ATT:

Equation 11:

$$S_{1bl}=S_{1GM}=0$$

For ATT ≤ t < ATT + LD:

Equation 12:

$$S_{1bl}\left( t \right)=2\alpha M_{0}CBF\frac{1}{{R'}_{1bl}}e^{-R_{1bl}\mathrm{ATT}}\left( 1-e^{-{R^{'}}_{1bl}(t-ATT)} \right)$$

Equation 13:

$$S_{1GM}\left( t \right)=2\alpha M_{0}CBFe^{-R_{1bl}\mathrm{ATT}}\left[ \frac{1}{R_{1GM}}\left( 1-e^{-R_{1GM}(t-ATT)} \right)- \frac{1}{{R'}_{1GM}}(1-e^{-{R^{'}}_{1GM}(t-ATT)}) \right]$$

For ATT + LD ≤ t:

Equation 14:

$$S_{1bl}\left( t \right)=2\alpha M_{0}CBFe^{-R_{1bl}ATT}\frac{e^{-{R^{'}}_{1bl}\left( t-ATT \right)}}{{R'}_{1bl}}\left( e^{R_{1bl}^{'}LD}-1 \right)$$

Equation 15:

$$S_{1GM}\left( t \right)=2\alpha M_{0}CBFe^{-R_{1bl}ATT}\left[ \frac{e^{-R_{1GM}(t-ATT)}}{R_{1GM}}\left( e^{R_{1GM}LD}-1 \right)-\frac{e^{-R_{1GM}^{'}(t-ATT)}}{R_{1GM}^{'}}\left( e^{R_{1GM}^{'}LD}-1 \right) \right]$$

After excitation at the start of the readout, signal evolves according to transverse relaxation. As label exchanges, its decay rate increases by ${\Delta R}_{2}$ due to a shorter T_2_ in the GM compartment. Similarly to Equations 4 and 5, the addition of K_bl🡪GM_ to the transverse relaxation rates reflects the apparent faster/slower T2 decay in the blood/GM compartments respectively as a result of exchange:

Equation 16:

$${R'}_{2bl}= R_{2bl}+K_{bl\to GM}$$

Equation 17:

$${\Delta R}_{2}= R_{2GM}-R_{2bl}$$

Equation 18:

$${\Delta R}_{2}'= R_{2GM}+K_{bl\to GM}-R_{2bl}$$

The total signal after the excitation pulse at a given TE is comprised of two parts:

Equation 19:

$$S_{X}=S_{2A}+S_{2B}$$

Where S_2A_ is the signal that is already in the voxel at the time of excitation, and S_B_ is the signal that did not yet arrive in the voxel at time of excitation, but exchanges into the compartment during the TE. Given this, S_A_ takes the form:

Equation 20:

$$S_{2Abl}\left( TE \right)=S_{1bl}e^{-R_{2bl}^{'}TE}$$

Equation 21:

$$S_{2AGM}\left( TE \right)=S_{1bl}{(1-e}^{-K_{bl\to GM}TE})e^{-R_{2GM}TE}+S_{1GM}e^{-R_{2GM}TE}$$

And S_2B_, in integral form is:

Equation 22:

$$S_{2Bbl}\left( TE \right)=2M_{0}CBF\int_{0}^{TE} c_{2}\left( t^{'} \right)r_{bl\to GM}\left( {TE-t}^{'} \right)m_{2bl}\left( TE-t^{'} \right)ⅆt^{'}$$

Equation 23:

$$S_{2BGM}\left( TE \right)=2M_{0}CBF\int_{0}^{TE} c_{2}\left( t^{'} \right)(1-r_{bl\to GM}\left( {TE-t}^{'} \right))m_{2GM}\left( TE-t^{'} \right)ⅆt'$$

With the residue function r_bl🡪GM_(t) defined as before, and m_2X_(t) now the T_2_ relaxation of the corresponding compartment. For this part of the equations, we must further differentiate subcases depending on the arrival of the signal bolus in the voxel. Indeed, the signal may not have reached the voxel at the point of excitation, but transport and exchange keep occurring after excitation and this would contribute to the signal. Our previous boundaries for c_1_(t) become our three initial cases, and additional boundaries arise depending on the advancement of the bolus in the time after the excitation. The input function therefore becomes:

Case 1: t < ATT

Equation 24:

$$c_{2}\left( TE \right)=\left\{ \begin{aligned} 0, TE<ATT-t \\ \alpha e^{-R_{1bl}t}e^{-R_{2bl}TE}, ATT-t\leq TE \end{aligned} \right.$$

Case 2: ATT ≤ t < LD + ATT

Equation 25:

$$c_{2}\left( TE \right)= \alpha e^{-R_{1bl}ATT}$$

Case 3: LD + ATT ≤ t

Equation 26:

$$c_{2}\left( TE \right)= 0$$

And the solution becomes :

Case 1: t < ATT

For TE < ATT – t:

Equation 27:

$$S_{2Bbl}=S_{2BGM}=0$$

For ATT – t ≤ TE < ATT + LD – t:

Equation 28:

$$S_{2Bbl}(TE)=2\alpha M_{0}CBFe^{-R_{1bl}t}e^{-R_{2bl}^{'}TE}\frac{1}{K_{bl\to GM}}(e^{K_{bl\to GM}TE}-e^{K_{bl\to GM}(ATT-t)})$$

Equation 29:

$$S_{2BGM}(TE)=2\alpha M_{0}CBFe^{-R_{1bl}t}e^{-R_{2GM}TE}\left[ \frac{1}{\Delta R_{2}}\left( e^{\Delta R_{2}TE}-e^{\Delta R_{2}\left( ATT-t \right)} \right)-\frac{e^{{-K}_{bl\to GM}TE}}{\Delta R_{2}^{'}}(e^{\Delta R_{2}^{'}TE}-e^{\Delta R_{2}^{'}\left( ATT-t \right)}) \right]$$

For ATT + LD – t ≤ TE:

Equation 30:

$$S_{2Bbl}(TE)=2\alpha M_{0}CBFe^{-R_{1bl}t}e^{-R_{2bl}^{'}TE}\frac{e^{K_{bl\to GM}\left( ATT-t \right)}}{K_{bl\to GM}}(e^{K_{bl\to GM}LD}-1)$$

Equation 31:

$$S_{2BGM}(TE)=2\alpha M_{0}CBFe^{-R_{1bl}t}e^{-R_{2GM}TE}\left[ \frac{e^{\Delta R_{2}\left( ATT-t \right)}}{\Delta R_{2}}\left( e^{\Delta R_{2}LD}-1 \right)-\frac{e^{\Delta R_{2}^{'}\left( ATT-t \right)}e^{-K_{bl\to GM}TE}}{\Delta R_{2}^{'}}(e^{\Delta R_{2}^{'}LD}-1) \right]$$

Case 2: ATT ≤ t < LD + ATT

For TE < ATT + LD – t :

Equation 32:

$$S_{2Bbl}(TE)=2\alpha M_{0}CBFe^{-R_{1bl}ATT}\frac{e^{-R_{2bl}^{'}TE}}{K_{bl\to GM}}(e^{K_{bl\to GM}TE}-1)$$

Equation 33:

$$S_{2BGM}(TE)=2\alpha M_{0}CBFe^{-R_{1bl}ATT}e^{-R_{2GM}TE}\left[ \frac{1}{\Delta R_{2}}\left( e^{\Delta R_{2}TE}-1 \right)-\frac{e^{-K_{bl\to GM}TE}}{\Delta R_{2}^{'}}(e^{\Delta R_{2}^{'}TE}-1) \right]$$

For ATT + LD – t ≤ TE:

Equation 34:

$$S_{2Bbl}(TE)=2\alpha M_{0}CBFe^{-R_{1bl}ATT}\frac{e^{-R_{2bl}^{'}TE}}{K_{bl\to GM}}(e^{K_{bl\to GM}(ATT-t+LD)}-1)$$

Equation 35:

$$S_{2BGM}(TE)=2\alpha M_{0}CBFe^{-R_{1bl}ATT}e^{-R_{2GM}TE}\left[ \frac{1}{\Delta R_{2}}\left( e^{\Delta R_{2}\left( ATT-t+LD \right)}-1 \right)-\frac{e^{-K_{bl\to GM}TE}}{\Delta R_{2}^{'}}(e^{\Delta R_{2}^{'}\left( ATT-t+LD \right)}-1) \right]$$

Case 3: LD + ATT ≤ t

Equation 36:

$$S_{2Bbl}=S_{2BGM}=0$$

**3-compartment model solution**

The 3-compartment model can be solved in a similar manner. For this, integral equations for the signal before excitation are:

Equation 37:

$$S_{1bl}\left( t \right)=2M_{0}CBF\int_{0}^{t} c_{1}\left( t^{'} \right)r_{bl\to GM}\left( {t-t}^{'} \right)r_{bl\to CSF}\left( {t-t}^{'} \right)m_{1bl}\left( t-t^{'} \right)ⅆt^{'}$$

Equation 38 :

$$S_{1GM}\left( t \right)=2M_{0}CBF\int_{0}^{t} c_{1}\left( t^{'} \right)(1-r_{bl\to GM}\left( {t-t}^{'} \right))m_{1GM}\left( t-t^{'} \right)ⅆt'$$

Equation 39 :

$$S_{1CSF}\left( t \right)=2M_{0}CBF\int_{0}^{t} c_{1}\left( t^{'} \right)(1-r_{bl\to CSF}\left( {t-t}^{'} \right))m_{1CSF}\left( t-t^{'} \right)ⅆt'$$

With magnetization relaxation and input functions unchanged. The residue function r_bl🡪(GM,CSF)_ also takes the same form as previously. Therefore, the GM and CSF functions are the same as for the 2-compartment model, and only the blood function changes to include transport to both the GM and CSF compartments simultaneously. To account for this, the variable R’_1b_ is changed to:

Equation 40:

$${R'}_{1bl}= R_{1bl}+K_{bl\to GM}+K_{bl\to CSF}$$

And R’_1(GM,CSF)_ are unchanged. By using this change of variable, all equations are the same as the 2-compartment model (eq. 11-15).

The same can be applied for the equations after excitation. R’_2bl_ becomes:

Equation 41:

$${R'}_{2bl}= R_{2bl}+K_{bl\to GM}+K_{bl\to CSF}$$

With this, the equations for S_2AX_ remain in the same form as for the 2-compartment model (eq. X and Y for blood and GM/CSF respectively).

For S_2B_, the equations in their integral form are:

Equation 42:

$$S_{2Bbl}\left( TE \right)=2M_{0}CBF\int_{0}^{TE} c_{2}\left( t^{'} \right)r_{bl\to GM}\left( {TE-t}^{'} \right)r_{bl\to CSF}\left( {TE-t}^{'} \right)m_{2bl}\left( TE-t^{'} \right)ⅆt^{'}$$

Equation 43:

$$S_{2BGM}\left( TE \right)=2M_{0}CBF\int_{0}^{TE} c_{2}\left( t^{'} \right)(1-r_{bl\to GM}\left( {TE-t}^{'} \right))m_{2GM}\left( TE-t^{'} \right)ⅆt'$$

Equation 44:

$$S_{2BCSF}\left( TE \right)=2M_{0}CBF\int_{0}^{TE} c_{2}\left( t^{'} \right)(1-r_{bl\to CSF}\left( {TE-t}^{'} \right))m_{2CSF}\left( TE-t^{'} \right)ⅆt'$$

Again, the input and magnetization relaxation functions are unchanged, and the solution to these equations is the same for the GM and CSF compartments, so they will not be repeated here. However, minor changes to the blood compartment equations appear because of the dual pathways for water exchange into both the GM and CSF compartments.

The solution for the blood compartment is:

Case 1: t < ATT

For TE < ATT – t:

Equation 45:

$$S_{2Bbl}=0$$

For ATT – t ≤ TE < ATT + LD – t:

Equation 46:

$$S_{2Bbl}(TE)=2\alpha M_{0}CBFe^{-R_{1bl}t}e^{-R_{2bl}^{'}TE}\frac{1}{{(K}_{bl\to GM}+K_{bl\to CSF})}(e^{{(K}_{bl\to GM}+K_{bl\to CSF})TE}-e^{{(K}_{bl\to GM}{+K}_{bl\to CSF})(ATT-t)})$$

For ATT + LD – t ≤ TE:

Equation 47:

$$S_{2Bbl}(TE)=2\alpha M_{0}CBFe^{-R_{1bl}t}e^{-R_{2bl}^{'}TE}\frac{e^{{(K}_{bl\to GM}+K_{bl\to CSF})\left( ATT-t \right)}}{{(K}_{bl\to GM}+K_{bl\to CSF})}(e^{{(K}_{bl\to GM}+K_{bl\to CSF})LD}-1)$$

Case 2: ATT ≤ t < LD + ATT

For TE < ATT + LD – t :

Equation 48:

$$S_{2Bbl}(TE)=2\alpha M_{0}CBFe^{-R_{1bl}ATT}\frac{e^{-R_{2bl}^{'}TE}}{{(K}_{bl\to GM}{+K}_{bl\to CSF})}(e^{{(K}_{bl\to GM}{+K}_{bl\to CSF})TE}-1)$$

For ATT + LD – t ≤ TE:

Equation 49:

$$S_{2Bbl}(TE)=2\alpha M_{0}CBFe^{-R_{1bl}ATT}\frac{e^{-R_{2bl}^{'}TE}}{{(K}_{bl\to GM}{+K}_{bl\to CSF})}(e^{{(K}_{bl\to GM}{+K}_{bl\to CSF})(ATT-t+LD)}-1)$$

Case 3: LD + ATT ≤ t

Equation 50:

$$S_{2Bbl}=0$$

**Supplementary figures
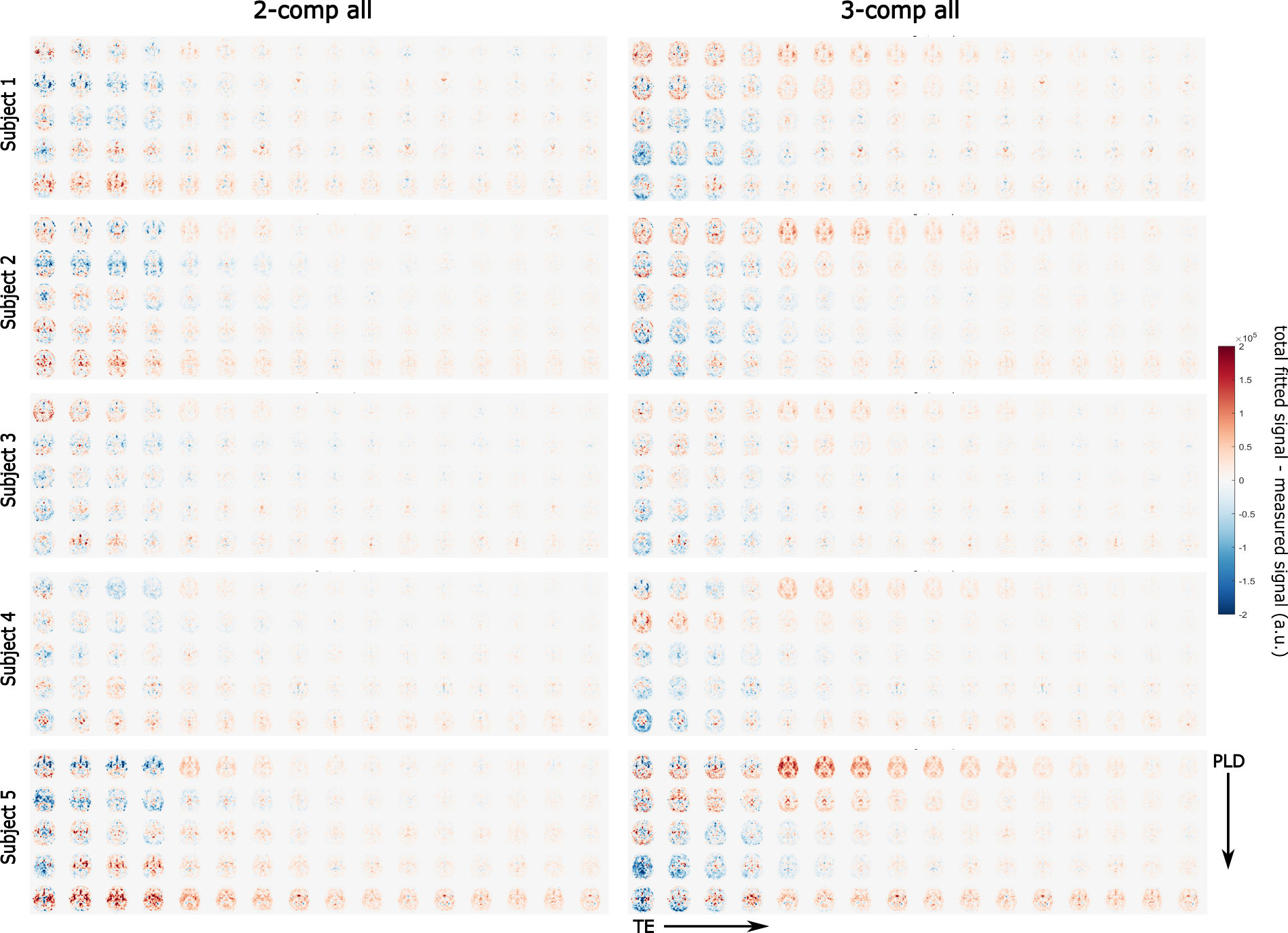
**

**Figure S1.** Single slice (15) Residual map of the difference between the total fitted signal and the measured signal at each TE/PLD for both the 2-comp all and 3-comp all models, for all subjects.

**Figure S2** (video uploaded separately). Whole-brain signal fractions in the blood, GM, and CSF for an artificial bolus of LD = 3s at PLD = 0-5s in subject 5.

**
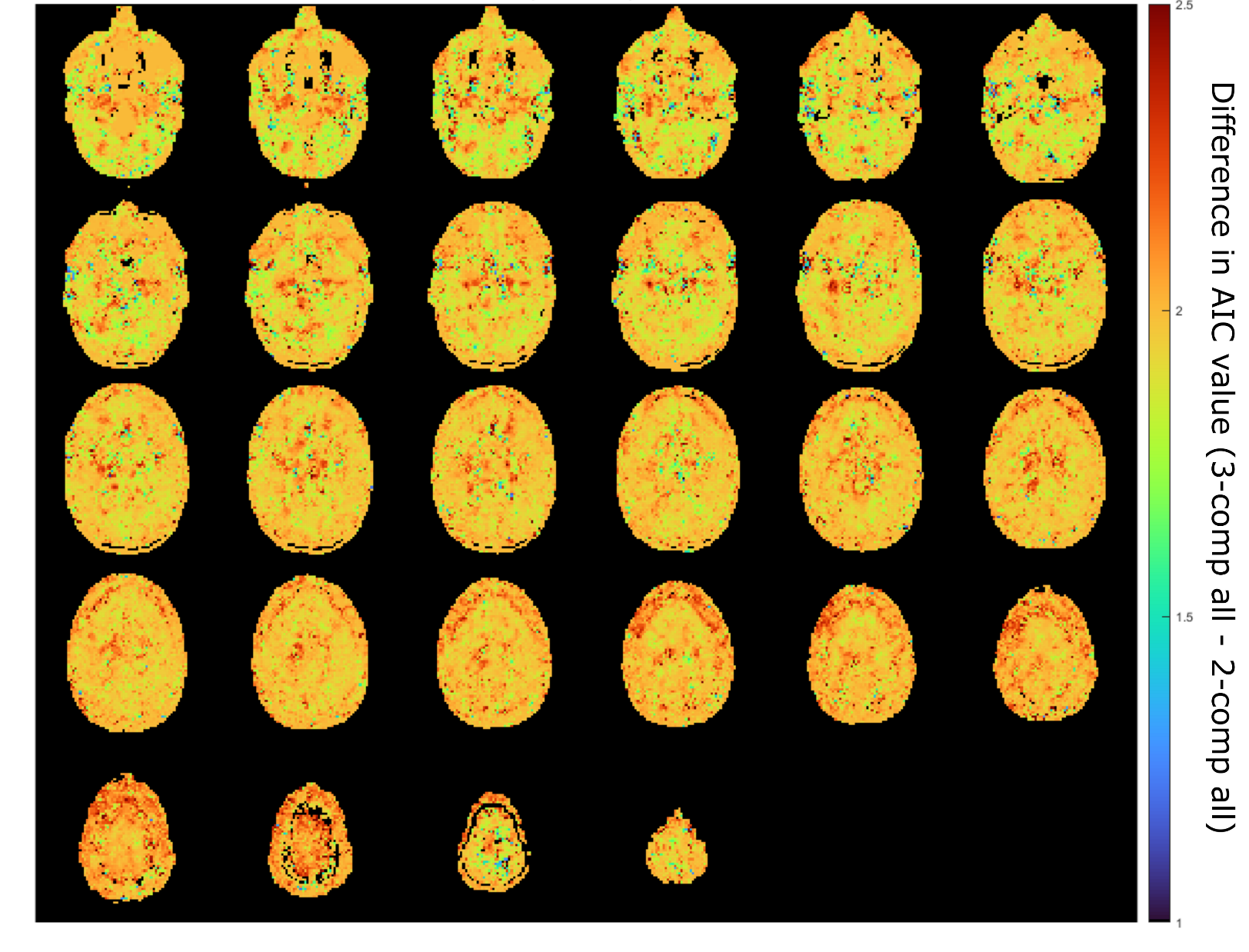
**

**Figure S3.** Whole brain maps of the difference in AIC values between the 3 compartment model (3-comp all) and 2 compartment model (2-comp-all) in a single subject.

**
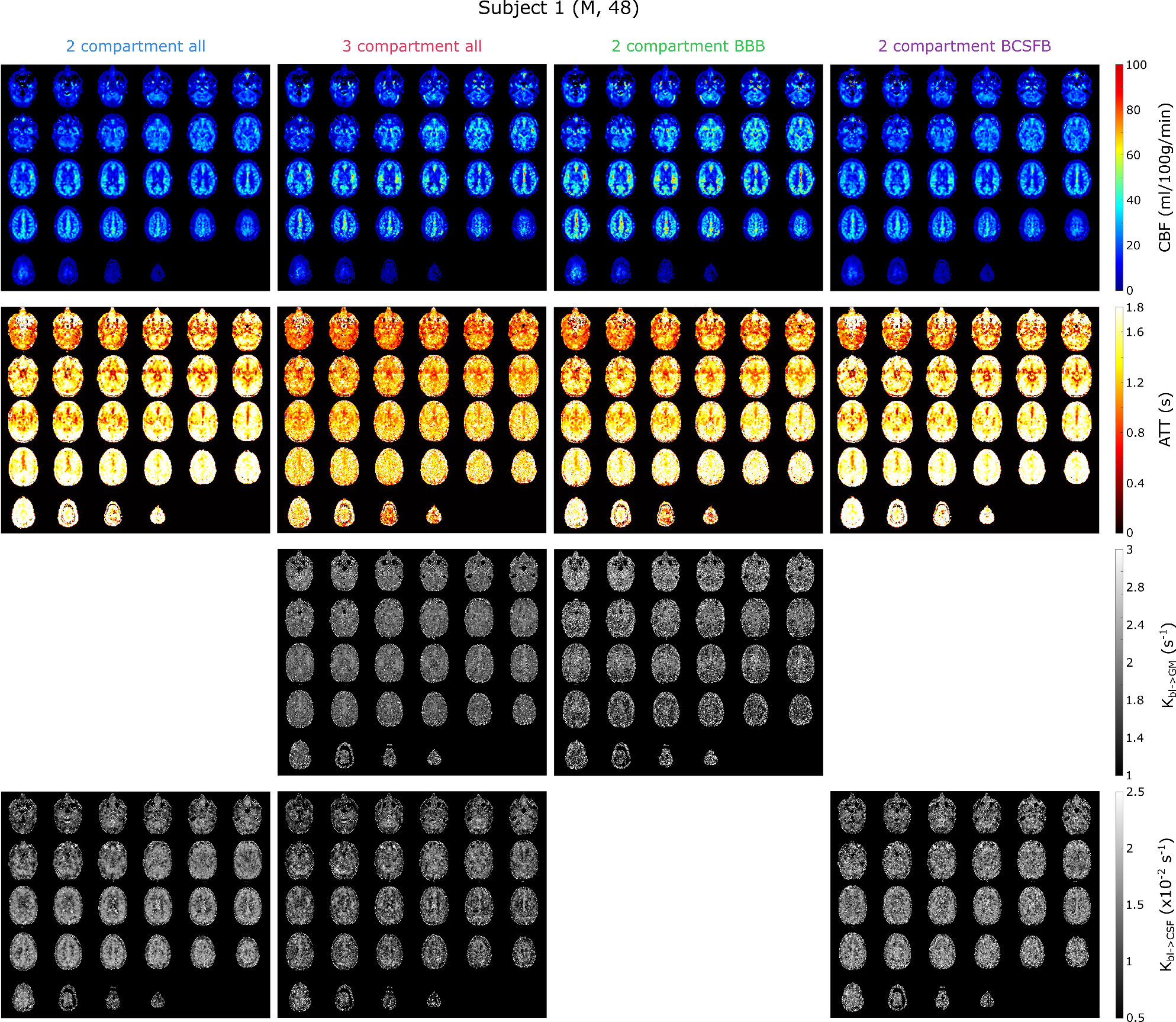
**

**Figure S4.** Whole-brain parameter maps for all models in subject 1 (M, 48).

**
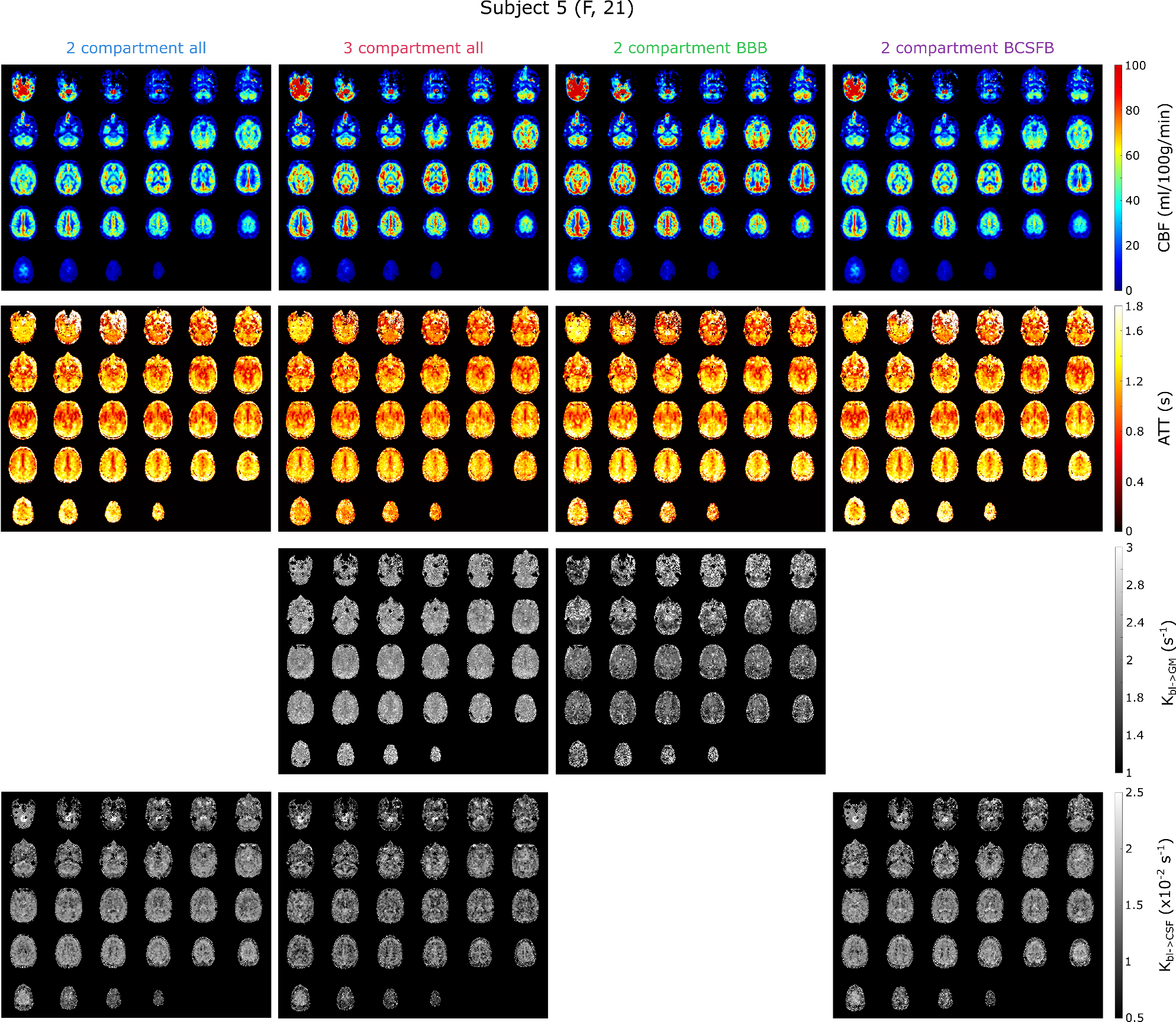
**

**Figure S5.** Whole-brain parameter maps for all models in subject 5 (F, 21).

**
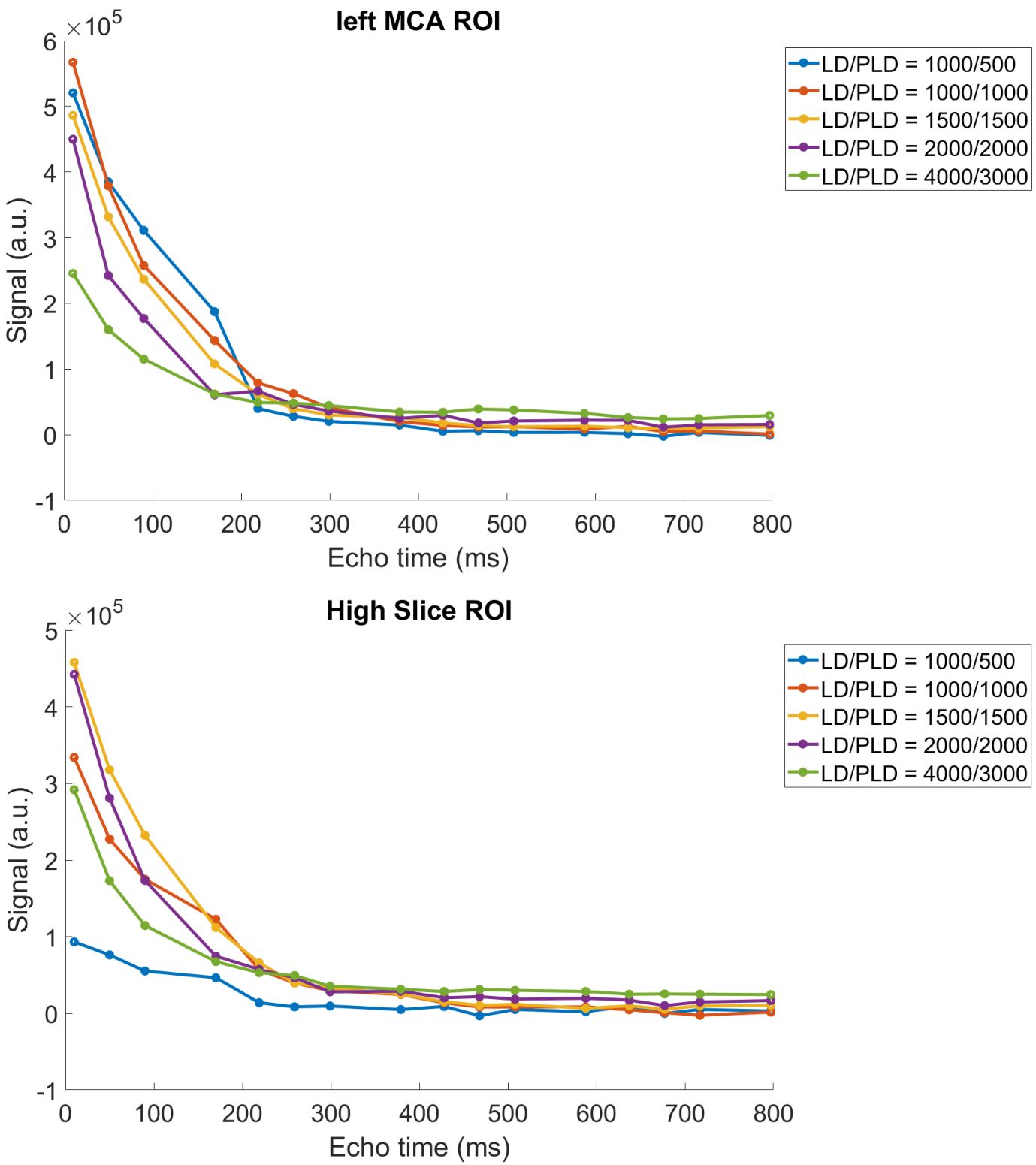
**

**Figure S6.** Average ASL signal in the left MCA and high slice ROIs, as a function of TE, for all LD/PLD pairs.

**
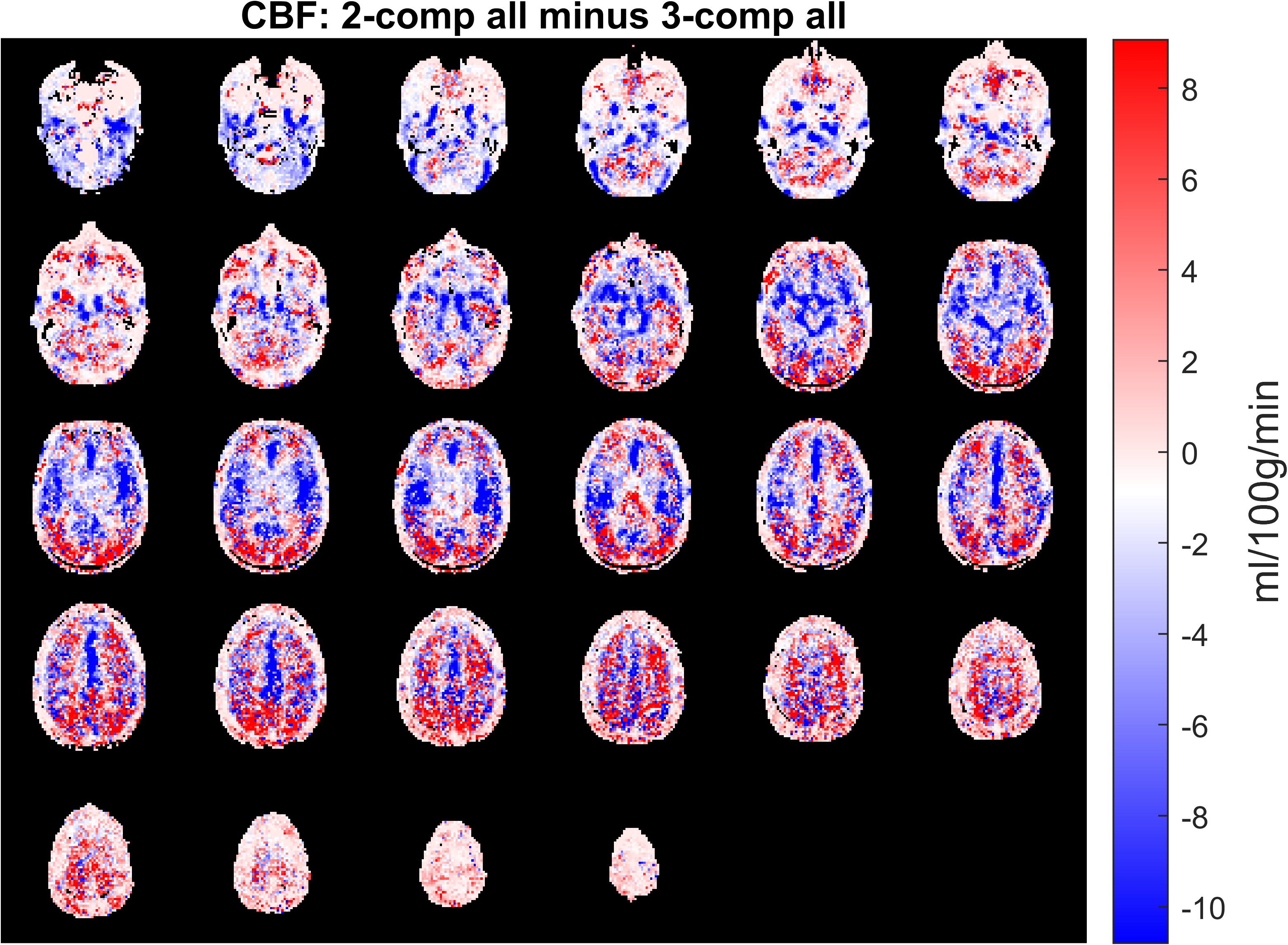
**

**Figure S7.** CBF difference map (2-comp all minus 3-comp all) for subject 1.

**
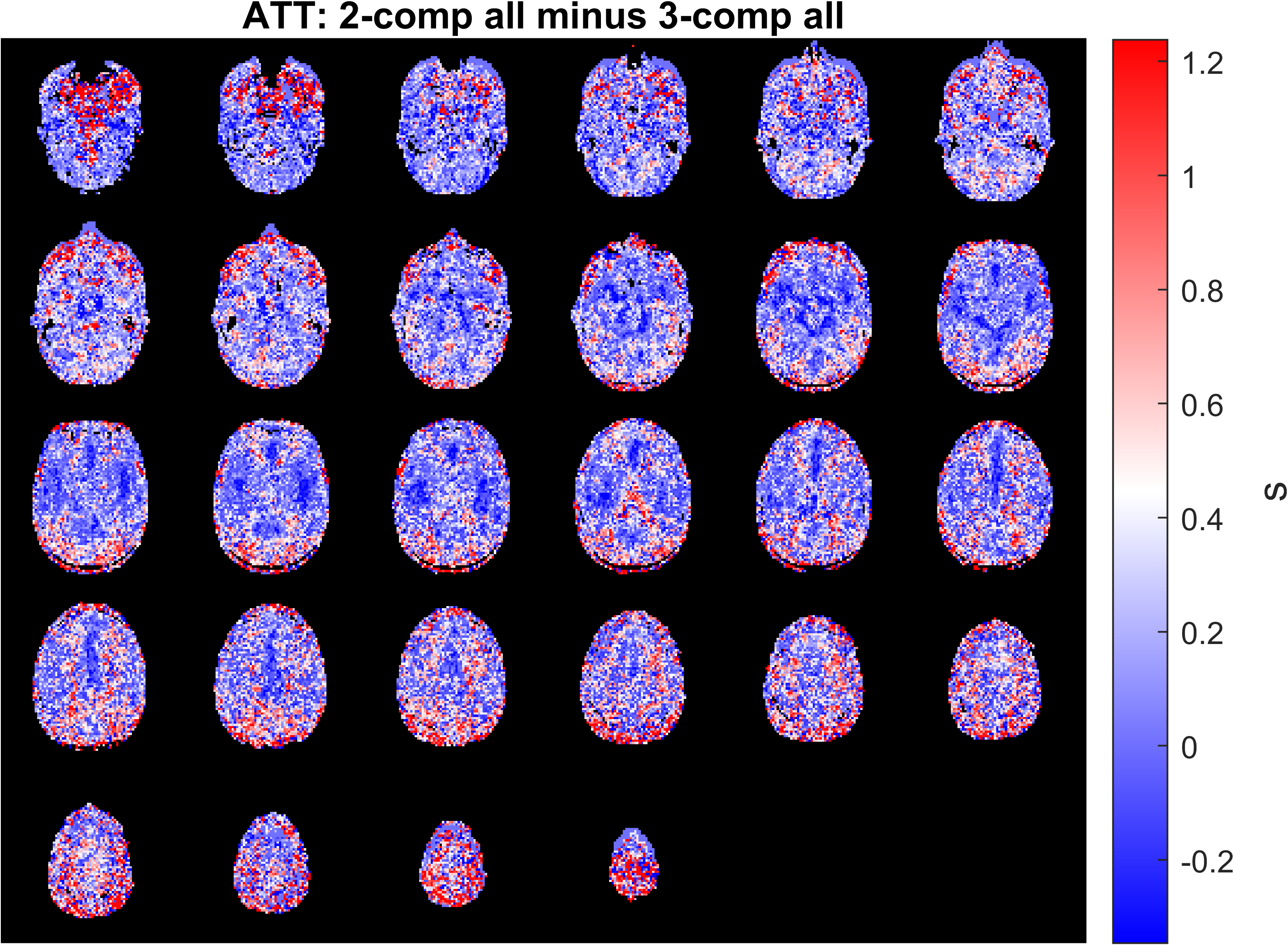
**

**Figure S8.** ATT difference map (2-comp all minus 3-comp all) for subject 1.

**
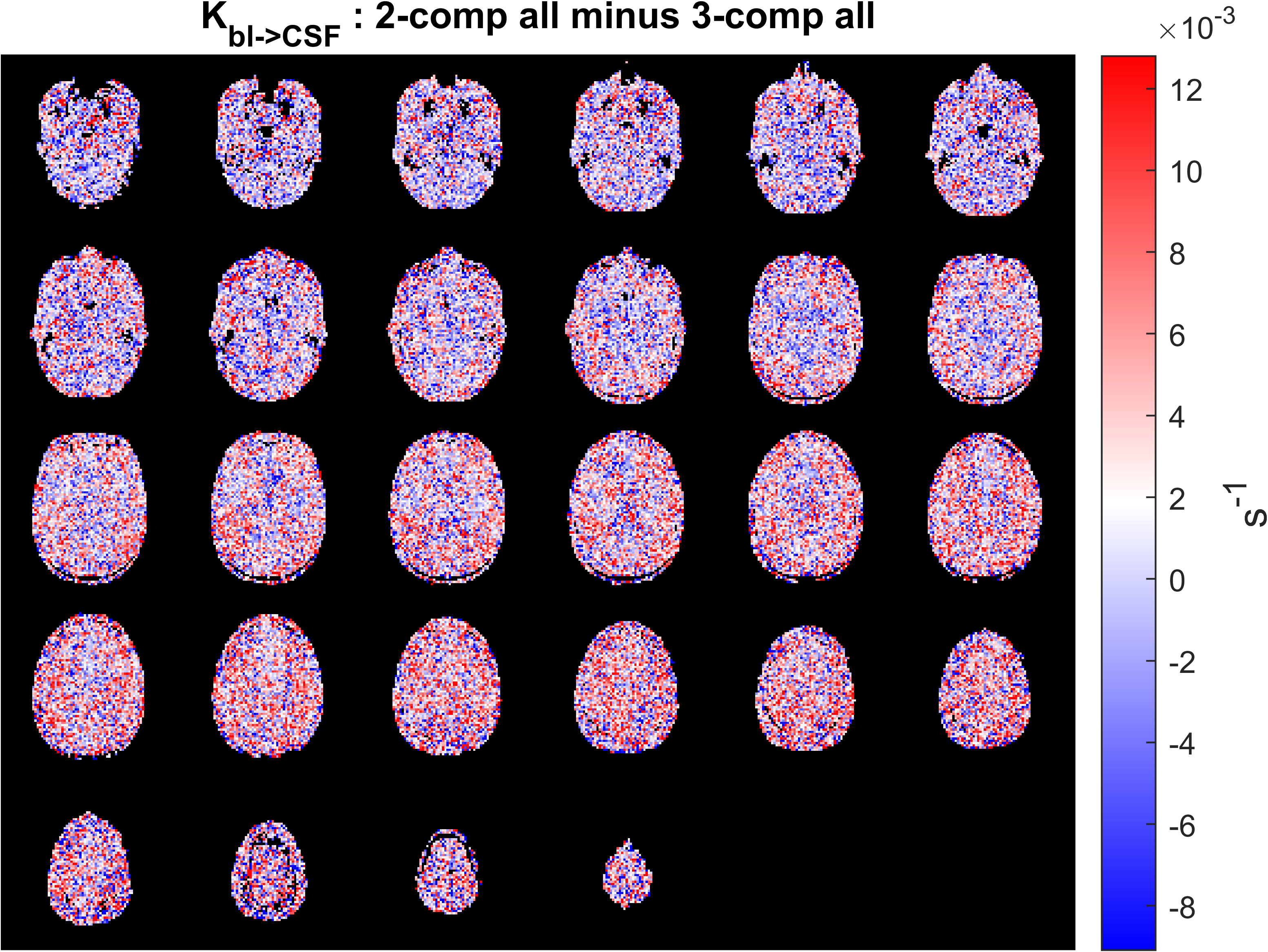
**

**Figure S9.** K_bl->CSF_ difference map (2-comp all minus 3-comp all) for subject 1.

**
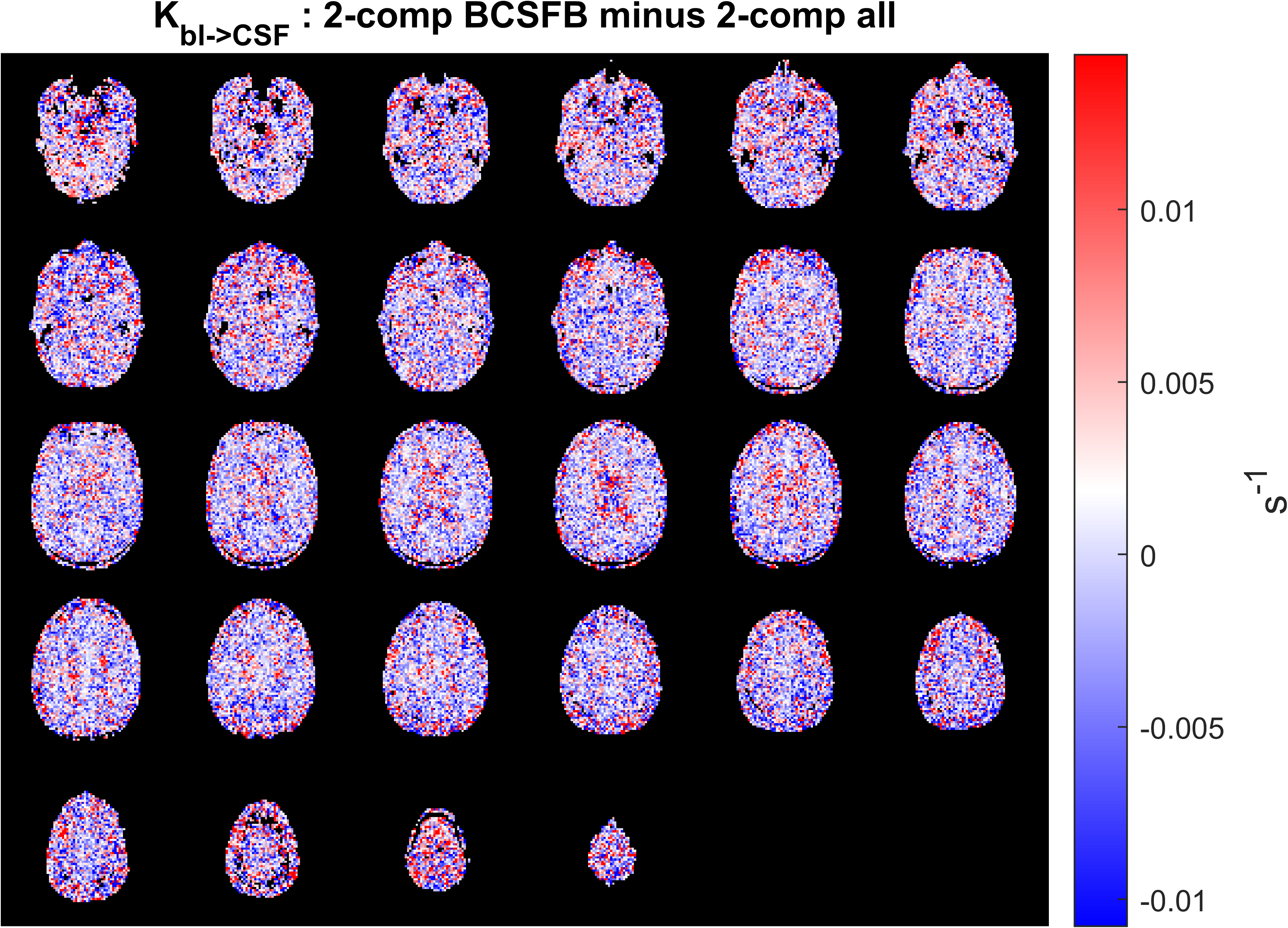
**

**Figure S10.** K_bl->CSF_ difference map (2-comp BCSFB minus 2-comp all) for subject 1.

**
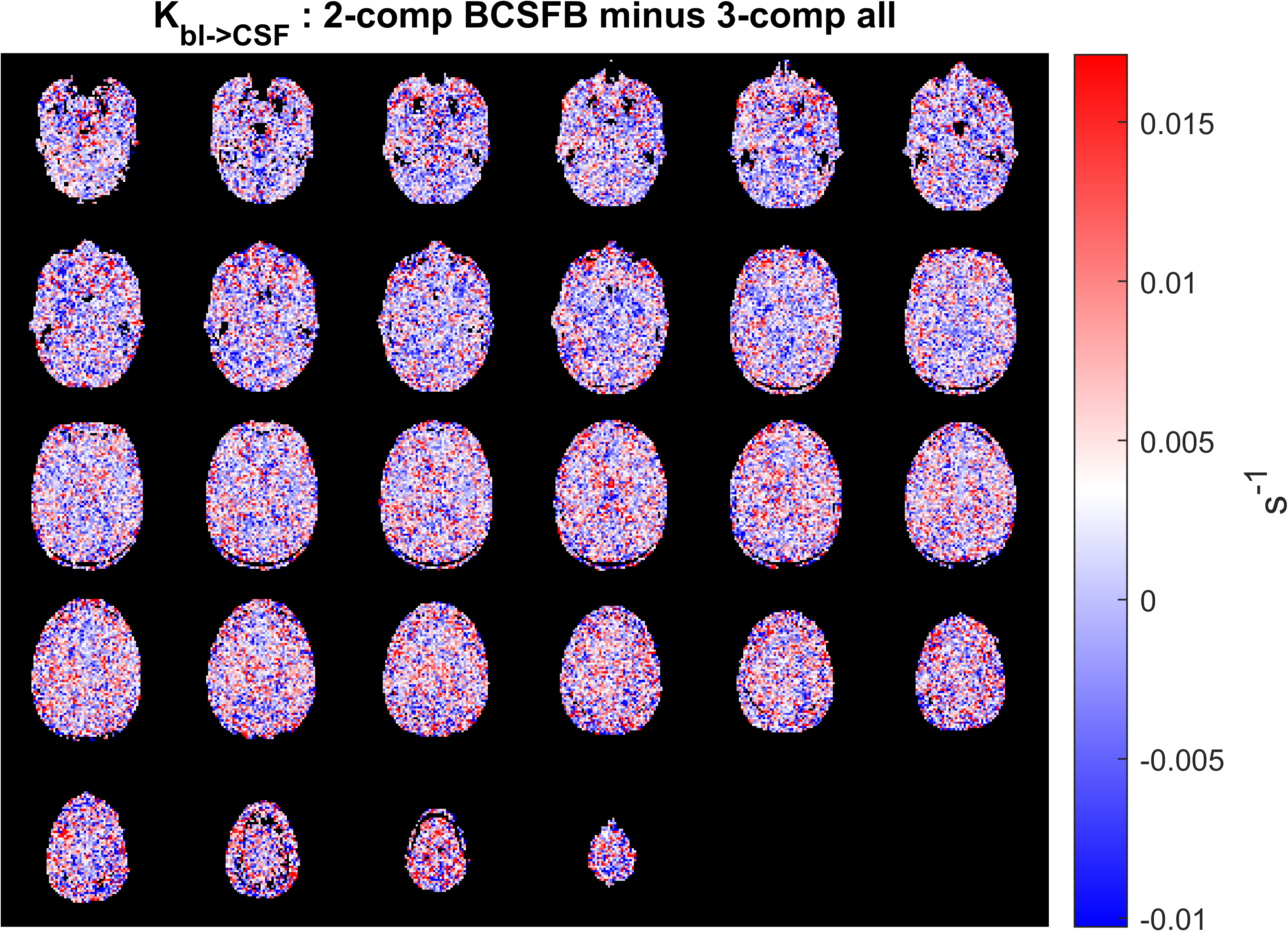
**

**Figure S11.** K_bl->CSF_ difference map (2-comp BCSFB minus 3-comp all) for subject 1.

**
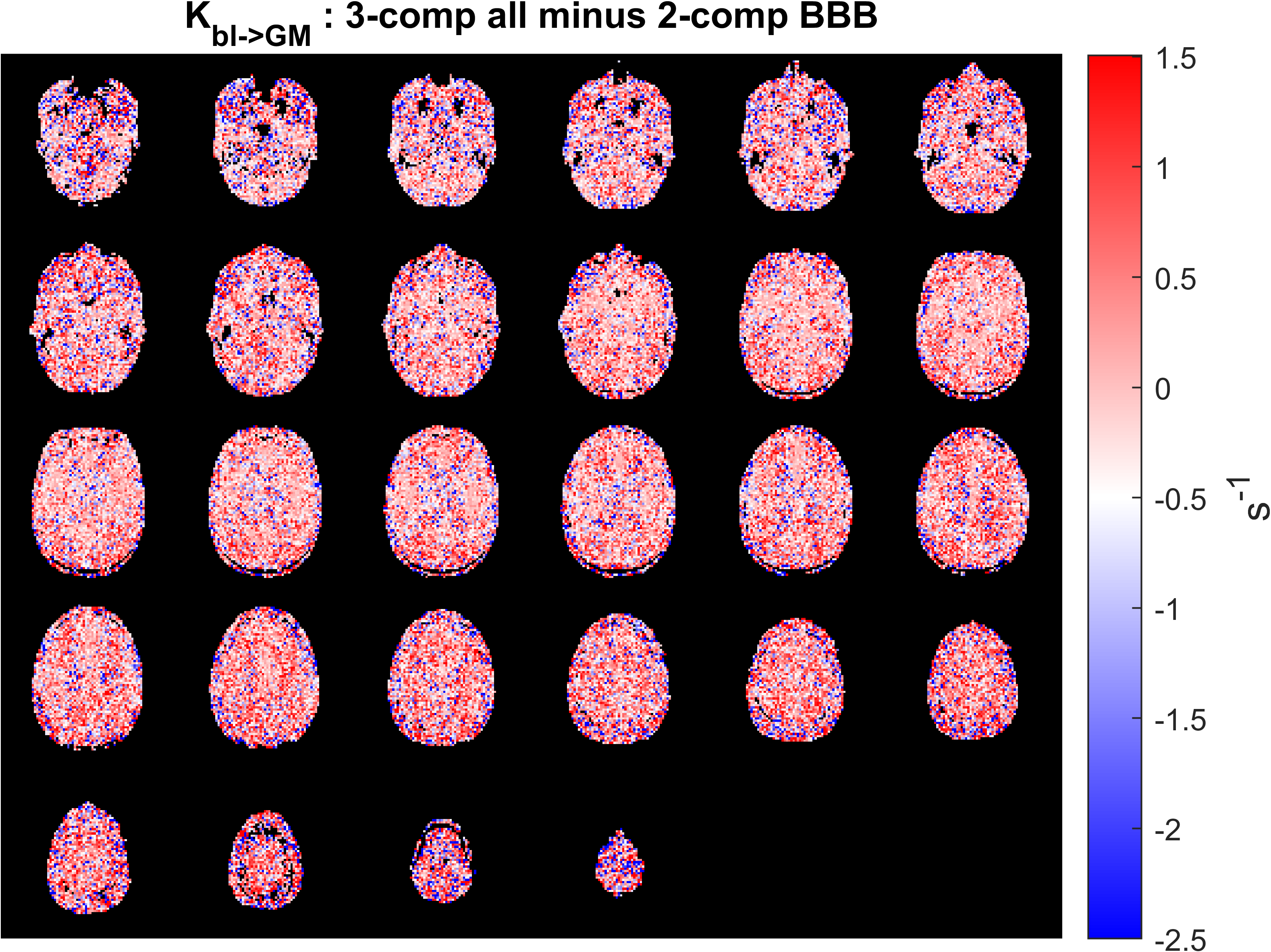
**

**Figure S12.** K_bl->GM_ difference map (3-comp all minus 2-comp BBB) for subject 1.
